# ELAPOR1 regulates VPS54-mediated GARP complex formation and proacrosomal vesicle fusion during spermatogenesis

**DOI:** 10.7150/thno.131535

**Published:** 2026-03-25

**Authors:** Chen Ding, Tingting Liang, Mina Yang, Lingbin Wang, Xuying Yin, Guoming Li, Zhaoxiang Wang, Jiahui Guo, Lin Zhang, Jie Yang, Xiaoyu Xia, Yanyan Xu, Junling Liu, Haojie Jiang

**Affiliations:** 1 Department of Biochemistry and Molecular Cell Biology, Shanghai Jiao Tong University School of Medicine, Shanghai, China.; 2 Department of Histo-Embryology, Genetics and Developmental Biology, Shanghai Jiao Tong University School of Medicine, Shanghai, China.; 3 Shanghai Key Laboratory of Reproductive Medicine, Shanghai Jiao Tong University School of Medicine, Shanghai, China.; 4 Department of Laboratory Medicine, Ruijin Hospital, Shanghai Jiao Tong University School of Medicine, Shanghai, China.; 5 Collaborative Innovation Center of Hematology, Shanghai Jiao Tong University School of Medicine, Shanghai, China.

**Keywords:** infertility, globozoospermia, spermatogenesis, acrosome, ELAPOR1

## Abstract

**Rationale:**

Acrosomal developmental defects are associated with severe sperm morphogenetic abnormalities and male infertility; however, the specific molecular determinants underlying these defects remain poorly defined. Endosome/lysosome-associated apoptosis and autophagy regulator 1 (ELAPOR1) is important for cellular membrane dynamics and organelle functions, indicating that it plays an essential role in spermatogenesis, which needs further investigation. This study aimed to explore the specific role of ELAPOR1 in spermatogenesis and male fertility.

**Methods:**

Single-cell RNA-sequencing datasets from human testes were obtained to investigate the expression of *ELAPOR1* in specific cell types. An *Elapor1* germ cell-specific knockout mouse line (*Elapor1^cKO^*) was established, and the morphology and function of the testis and sperm were assessed through breeding capacity, fertilization capacity, testicular histology, sperm staining, concentration and motility, immunofluorescence, quantitative PCR, immunoblotting, and transmission electron microscopy analyses. Moreover, mass spectrometry and enrichment analyses were employed to identify the proteins that interact with ELAPOR1. The interactions were verified through proximity labeling, coimmunoprecipitation, and immunofluorescence staining.

**Results:**

ELAPOR1 is highly expressed and its protein colocalized with the acrosome during the early stage of acrosome formation. *Elapor1^cKO^* mice produced deformed sperm with decreased concentration, impaired motility, and defective fertilization capacity. Moreover, ELAPOR1 deficiency led to impaired fusion of proacrosomal vesicles. Mechanistically, ELAPOR1 functioned in regulating the transport of the Golgi and early endosome-related vesicles. It interacted with VPS54 and affected VPS54-associated assembly of the GARP complex in the testis.

**Conclusions:**

Our findings reveal the essential role of ELAPOR1 in acrosome formation during spermatogenesis and male fertility. ELAPOR1 potentially influences the trafficking, integration, and fusion of proacrosomal vesicles through VPS54-mediated GARP complex assembly. These findings provide novel insights into the interaction of the ELAPOR1-GARP complex in acrosome-related reproductive failure, suggesting that *Elapor1* deficiency or mutation could be considered a potential genetic risk factor for human infertility.

## Introduction

Spermatogenesis represents a sophisticated and essential biological process that is crucial for the production of mature spermatozoa. This intricate process involves the development of specialized cellular components, including a functional acrosome for egg penetration, a motile flagellum for propulsion, and a highly condensed nucleus containing genetic material, all of which are indispensable for successful fertilization. The biogenesis of the acrosome is a critical and meticulously regulated process that generates a reservoir of glycosylated digestive enzymes, including hyaluronidase and acrosin, which are crucial for penetrating the protective layers of the oocyte. Starting in the early stage of spermiogenesis, the essential organelle develops through a precisely orchestrated sequence of events that can be systematically divided into four distinct developmental phases: 1) the Golgi phase—proacrosomal vesicle generation; 2) the cap phase—polar cap formation; 3) the acrosome phase—acrosomal expansion; and 4) the maturation phase—the final structural maturation 1-3. The acrosome biogenesis process is initiated by the precise accumulation of Golgi-derived proacrosomal vesicles in the nuclear indentation of developing spermatids. Through highly regulated membrane fusion mechanisms, these vesicles coalesce into one large acrosomal vesicle. This nascent structure then establishes a stable connection to the nuclear envelope through a specialized cytoskeletal basement called the acroplaxome, which serves as a structural scaffold for acrosome-nucleus anchoring 1. Subsequent acrosomal growth occurs through continuous vesicle fusion, leading to its expansion over the anterior nuclear pole 1-3. Despite detailed descriptions of the morphological changes that occur during acrosome development, the fundamental molecular mechanism regulating this process remains poorly explored and elusive and represents an important area for future investigation.

The functional competence of spermatozoa is critically dependent on proper acrosome formation, with developmental defects ultimately compromising fertility. Globozoospermia, a rare (< 0.1% infertility prevalence) but clinically significant reproductive disorder among acrosome-related conditions characterized by rounded sperm nuclei and malformed or completely absent acrosomes, exhibits strong genetic associations based on the current evidence 4-9. Mouse models have provided significant insights into the molecular mechanisms underlying acrosome biogenesis and globozoospermia, which can be divided into two critical aspects: vesicle dynamics and acrosomal-nuclear interactions. In one aspect, proteins involved in the proper formation, intracellular trafficking, and targeted fusion of proacrosomal vesicles are essential during acrosome development. These proteins include GBA2 10, ENPL 11, PICK1 12, GOPC 13, SMAP2 14, VPS54 15, AGFG1 16, GM130 17, AU040320 18, SIR1 19, and ATG7 20, each of which play critical roles in the integration of acrosomal granules. In addition, proteins that regulate precise acrosomal positioning and nuclear coverage are essential for normal acrosome function. These proteins include ZPBP1/2 21, CSK22 22, SACA1 23, and D19L2 24, which participate in acrosome attachment and anchoring. The identification and functional characterization of these diverse proteins underscore the complexity of acrosome biogenesis and the precisely regulated molecular pathways that can lead to globozoospermia when disrupted.

VPS54 functions to regulate endosome-to-Golgi retrograde transport that is essential for proper acrosome formation 15. The wobbler (*wr/wr*) mouse model carries the L967Q missense mutation in *Vps54*, resulting in defective spermiogenesis and progressive neurodegeneration 25. VPS54 is a distinct component of the Golgi-associated retrograde protein (GARP) complex that facilitates vesicle tethering and sorting by interacting with SNARE complexes and regulates the retrieval of the cation-independent mannose 6-phosphate receptor (CI-M6PR) and TGN46 26. Furthermore, leucine-to-glutamine replacement in *wr* mice disrupts the stability of the VPS54 structure and ultimately results in decreased protein abundance of VPS54 and the GARP complex, suggesting its crucial role in maintaining trans-Golgi network homeostasis and proper sorting of secretory cargos 27.

Endosome/lysosome-associated apoptosis and autophagy regulator (ELAPOR1), alternatively termed KIAA1324/EIG121/inceptor, is a single-pass type I membrane protein that is localized to multiple intracellular membranes. Its name derives from its initial molecular characterization, which reveals its localization to endosomes and lysosomes, as well as its regulatory role in autophagic processes 28. Moreover, recent studies have elucidated additional functional roles of ELAPOR1 in various cellular processes, including secretory granule maturation in gastric cells 29, clathrin-mediated endocytosis 30 and insulin signaling regulation 31. These diverse functions highlight the multifaceted roles of ELAPOR1 in terms of cellular membrane dynamics and organelle functions. Whole-body *Elapor1* knockout causes male infertility 29, 32, 33. However, the widespread tissue distribution of ELAPOR1 suggests potential interorgan crosstalk that may modulate testicular function during spermatogenesis, such as in the endocrine glands. Therefore, the use of whole-body knockout mouse strains may cause some confusion in determining the exact function of ELAPOR1 in spermatogenesis.

Here, we engineered germline-specific *Elapor1* knockout mice to elucidate the function of ELAPOR1 in male fertility given its widespread expression throughout the organism. Our findings reveal a phenotype characterized by globozoospermia and complete male infertility. Specifically, *Elapor1* conditional knockout in the male germline leads to the production of immotile, round-headed spermatozoa that lack an acrosomal structure. Developmental defects manifest as impaired proacrosomal vesicular fusion during early spermiogenesis, disorganized mitochondria, and deformed manchette structures. Through detailed analyses, we demonstrated that ELAPOR1 deficiency likely disrupts the sorting and recruitment of protein vesicles essential for acrosome formation. Collectively, the results of our study reveal that ELAPOR1 is an essential factor for male fertility in mice and reveal novel molecular mechanisms of VPS54-mediated GARP complex assembly underlying acrosome-related reproductive failure. These findings provide new insights into the molecular pathways that regulate acrosome biogenesis and their implications for male infertility.

## Materials and Methods

### Antibodies and reagents

The specific antibodies and reagents used are listed in Table S1.

### Animals

All the mice used were bred on a C57BL/6J genetic background. *Elapor1-floxed* mice (C57BL/6J-5330417C22Rik*^em1(flox)Cya^*) were constructed by Cyagen Biosciences (Suzhou, China). Briefly, CRISPR/Cas-mediated genome engineering was used to insert loxP sites flanking exons 3 and 6 of the ubiquitously expressed *Elapor1*-201 (ENSMUST00000048012) transcript. The deletion of this region should result in the loss of function of the mouse 5330417C22Rik gene.

*Elapor1^flox/-^; Stra8-Cre* (simplified as* Elapor1^cKO^*) mice were obtained by crossing *Elapor1^wt/flox^; Stra8-Cre* (simplified as *Elapor1^wt/cKO^*) males with *Elapor1^flox/flox^* (simplified as *Elapor1^flox^*) females. Genomic DNA extracted from tail tips was analyzed by PCR amplification to verify the deletion genotype. The primers used in this experiment are listed in Table S2.

The mice were housed in cages with individual ventilation (IVCs) under specific pathogen-free (SPF) conditions with controlled lighting (12:12 h light/dark cycle) and ad libitum access to autoclaved water and irradiated food. All animal experiments complied with the relevant ethical regulations and were conducted in accordance with international standards and guidelines that were approved by the Institutional Animal Care and Use Committee (IACUC) of Shanghai Jiao Tong University School of Medicine (Approval no. JUMC2023-093-A).

### Isolation of spermatogenic cells

Spermatogenic cells were isolated from the testes of 8-week-old male mice and subsequently subjected to immunofluorescence staining. Briefly, testes were collected, minced, and rinsed with PBS, after which the seminiferous tubule pieces were digested in an enzyme mixture (DMEM supplemented with 1.5 mg/mL of hyaluronidase, 0.5 mg/mL type IV collagenase, 0.25% trypsin-EDTA, and 0.1 mg/mL DNase I) for incubation (30 min, 34 °C) with gentle pipetting for several times. The digestion was terminated by adding an equal volume of a 3% BSA solution. After filtration through 40 μm PBS-saturated cell strainers, spermatogenic cells were obtained by centrifugation (400 × g, 5 min, 4 °C). Following the removal of the supernatant, the cells were washed three times with PBS for subsequent experiments.

### Fertility assessment

Each male mouse was paired with two age-matched *Elapor1^flox^* females that were at least 2 months old to generate offspring. For the assessment of fertility, each experimental male was continuously housed with two age-matched *Elapor1^flox^* female partners for 8 weeks, after which the timing of parturition and the neonatal cohort sizes were tracked.

### Protein extraction and immunoblot analysis

Tissues/cells were collected and homogenized in ice-cold cell lysis buffer (P0013; Beyotime Biotechnology, Shanghai, China) supplemented with a protease inhibitor cocktail (1:100) using a cryogenic grinder (Shanghai Jingxin, Shanghai, China) to extract total protein. The lysates were then centrifuged at 12,000 × *g* (20 min, 4 °C), after which the supernatants were collected and quantified using a BCA assay (20201ES86, Yeasen, Shanghai, China). Protein samples were separated by SDS‒PAGE (8‒12% gels) and subsequently transferred to PVDF membranes (Millipore Corporation, Massachusetts, USA, ISEQ00010). After blocking with 5% skim milk in TBST (Tris-buffered saline + 0.1% Tween-20) at RT for 1 h, the membranes were washed with TBST (3 × 10 min) and incubated with primary antibodies (4 °C, overnight) with continuous gentle inversion. After three washes with TBST the following day, the membranes were probed with secondary antibodies (diluted 1:5000-1:10000 in 5% skim milk) at RT for 1 h and then washed with TBST. Biotinylated proteins were detected with HRP-conjugated streptavidin (1:40,000; 35105ES60, Yeasen, Shanghai, China). Immunoreactive signals were detected using an ECL substrate kit (P10300, NCM Biotech, Suzhou, China), and images of the chemiluminescent signals were captured using an imaging system (5200, Tanon Technology, Shanghai, China). Band intensities were quantified using ImageJ (NIH, Washington DC, USA).

### Histological and immunofluorescence staining assays

Male mice were euthanized, and the morphology of the body and the testes were recorded. Testicular tissues were collected, washed with cold PBS and subsequently fixed with 4% PFA for 24-48 h at 4 °C. Following dehydration through a gradual ethanol series (70%-100%) and xylene, the tissues were embedded in paraffin. The tissues were sectioned into serial 5-μm slices (Leica RM2235, Wetzlar, Germany) and spread on slides precoated with poly-L-lysine. After deparaffinization and rehydration with xylene and a descending ethanol gradient (100%-70%), the sections were processed for routine hematoxylin and eosin (H&E), immunofluorescence, or periodic acid-Schiff (PAS) staining.

For hematoxylin and eosin (H&E) staining, the sections underwent strictly timed staining with hematoxylin (BA4097, Baso, Zhuhai, China; 3 min), acid-ethanol differentiation (1% HCl in 70% ethanol; 30 s), and bluing in distilled water before counterstaining with eosin (BA4098, Baso, China; 1 min). Following sequential dehydration through a graded ethanol series (80%-100%) and clearing in xylene, the sections were mounted with neutral balsam (36313ES60, Yeasen, Shanghai, China) and imaged using a bright-field microscope.

For immunofluorescence (IF) staining, the sections were stained with a tyramide signal amplification (TSA) staining kit (AFIHC034, AiFang Biological, Changsha, China). Briefly, the tissue sections were subjected to antigen retrieval in preheated 10 mM citrate buffer (pH 6.0) in a water bath (20 min, 95 °C), and then gradually cooled in air (1 h, RT). After three washes with PBS, a 3% H₂O₂ solution was added to the sections to quench endogenous peroxidase activity (15 min, RT). For background reduction, 3% BSA (36101ES80, Yeasen, Shanghai, China) in PBS was used to block nonspecific binding sites (1 h, RT). Following an overnight incubation with antibodies diluted in blocking buffer at 4 °C, the sections were rinsed with PBS and subsequently incubated with poly-HRP-conjugated antibodies (1 h, RT). Immunofluorescence signals were subsequently amplified using fluorescein-conjugated tyramine (10 min, RT). Finally, the sections were stained with FITC-PNA (1:20, 30 min) and DAPI (1 μg/mL, 10 min). The sections were rinsed with deionized water and sealed with Antifade Mounting Medium (36307ES25, Yeasen, Shanghai, China). Images were captured using a microscope (Zeiss, Oberkochen, Germany; Leica, Wetzlar, Germany; Nikon, Tokyo, Japan) with consistent laser power and gain settings across samples.

For periodic acid-Schiff (PAS) staining, the sections were stained with a PAS staining kit (C0142S, Beyotime, Shanghai, China). Briefly, tissue sections were incubated with a periodic acid solution (10 min, RT) and washed with distilled water for 5 min. Then, the sections were incubated with Schiff reagent (45 min, 37 °C) and washed with distilled water for 5 min. The sections underwent hematoxylin staining, acid ethanol differentiation, sequential dehydration, and mounting and were subsequently imaged as described previously.

### Sperm isolation and computer-assisted semen analysis (CASA)

Upon dissection, the cauda epididymis was immediately placed in prewarmed Tyrode's solution (37 °C, 5% CO₂) and cut carefully to allow sperm dispersion. After 30 min of incubation, the supernatant was collected for assessment. The sperm concentration and motility parameters were evaluated using a computer-assisted semen analysis (CASA) system (Hamilton Thorne Biosciences, MA, USA). For each biological replicate, ≥ 200 sperm tracks were analyzed across 10 random fields.

### Sperm staining

The sperm were isolated according to the procedure described previously and gently washed with prewarmed Tyrode's solution (3 × 5 min). Following centrifugation (1,000 × g, 10 min, RT) and resuspension in 4% paraformaldehyde (30 min, RT), the cells were spread on poly-L-lysine-precoated slides for fixation. The cells on the slides were air-dried and washed with PBS. Similar immunofluorescence assays were performed. The sperm were stained with FITC-PNA (1:20, 50 min), DAPI (1 μg/mL, 10 min), or MitoTracker (200 nM, 50 min).

The sperm were stained with a Giemsa staining kit (C0131, Beyotime, Shanghai, China), and the cells on the slides were immersed in freshly prepared working solution (1:20 dilution in PBS; 15 min, RT), followed by gentle rinsing with distilled water. After being sealed with neutral balsam (36313ES60, Yeasen, Shanghai, China), the sperm were imaged under a microscope (Zeiss, Oberkochen, Germany).

### Binding between sperm and eggs and *in vitro* fertilization (IVF)

Four-week-old female mice were superovulated with an intraperitoneal injection of 5 IU of pregnant mare serum gonadotropin (PMSG). Forty-eight hours later, injections of 5 IU of human chorionic gonadotropin (hCG) were administered to the same females. Cumulus-oocyte complexes (COCs) were harvested from the oviduct ampulla 14 hours after the hCG injection.

For the assessment of sperm-egg binding, capacitated caudal epididymal sperm were added to COCs and incubated for 30 min at 37 °C. The samples were subsequently fixed with 4% PFA for 15 min, rinsed three times with PBS, and then stained with Hoechst 33342 to visualize the bound sperm and ZP2 to visualize the zona pellucida.

For IVF, the eggs were transferred into an HTF medium (Aibei Biotechnology, Nanjing, China) containing sperm. After 5 h of incubation, the eggs were washed several times with HTF medium and transferred to KSOM medium (Aibei Biotechnology, Nanjing, China) for culture at 37 °C with 5% CO_2_. The proportion of two-cell embryos was evaluated at 24 h after insemination. Images were captured using a microscope (Nikon, Tokyo, Japan) with consistent laser power and gain settings across samples.

### Analysis of the acrosome reaction

As described previously, caudal epididymal sperm were collected and capacitated by incubation in HTF medium (2 h, 37 °C). Capacitated sperm were then incubated with the calcium ionophore A23187 (10 μM; HY-N6687, MedChemExpress, USA) at 37 °C for 30 min to induce the acrosome reaction. After being air-dried and washed with PBS, the sperm were placed onto poly-L-lysine-precoated slides, fixed with 4% PFA, and stained with PNA to assess acrosomal integrity.

### RNA extraction and quantitative real-time PCR analysis

Total RNA was extracted from homogenized testes using a cryogenic grinder (Jingxin, Shanghai, China) with RNA Extraction Reagent (R401, Vazyme Biotech Co., Ltd., Nanjing, China) according to the manufacturer's protocols. The integrity of the harvested RNA was verified by spectrophotometry using a Nanodrop instrument (Thermo Fisher Scientific, MA, USA) with an A260/A280 ratio > 1.9. cDNA was synthesized with a PrimeScript RT reagent Kit (RR036A, Takara, Japan) to reverse transcribe equal amounts of RNA from all samples. qPCR amplification was performed using a TB Green qPCR kit (RR820, Takara, Japan) on a LightCycler system (Roche Diagnostics). Relative mRNA expression was calculated through the efficiency-corrected 2^-ΔΔCt^ method with normalization to the *Gapdh* reference gene. The primers used in this experiment are listed in Table S2.

### Biotin tracer assay

Blood‒testis barrier permeability was examined with a biotin tracer using a previously described protocol 34. Briefly, anesthetized mice from each group were injected with 50 μL of Sulfo-NHS-LC-Biotin (10 mg/mL, HY-D0799, MedChemExpress), which was freshly diluted in PBS supplemented with 1 mM CaCl_2_. After 30 min, the testes were removed and embedded in OCT compound. Five-micron-thick cryosections were fixed with 4 % PFA for 20 min, rinsed with PBS, and subsequently blocked with 3% BSA for 1 h. The sections were then incubated with Alexa Fluor 488-conjugated streptavidin (35103ES60, Yeasen, Shanghai, China) at RT for 2 h. After the incubation, the sections were washed three times with PBS and stained with DAPI. After three washes with PBS, the sections were observed under a microscope (Nikon, Tokyo, Japan).

### Transmission electron microscopy (TEM)

Testicular tissues were dissected into small cubes (approximately 1 mm^3^) and immediately fixed with 4% glutaraldehyde in 0.2 M cacodylate buffer (4 °C, overnight). Following three washes, postfixation was performed using 2% osmium tetroxide (OsO₄) at 4 °C for 2 h. After dehydration in a graded ethanol series (50%-100%), the tissues were embedded in EPON resin. The samples were cut into ultrathin sections approximately 70 nm thick, collected on 200-mesh copper grids, and sequentially stained with 2% uranyl acetate followed by lead citrate to enhance image contrast. The samples were observed under a CM-120 transmission electron microscope (FEI Company, OR, USA).

### Mass spectrometry and enrichment analysis

Testicular lysates were immunoprecipitated using anti-ELAPOR1 antibodies, followed by pull-down with Protein A/G beads. An isotype-matched control IgG antibody was used as the negative control to confirm the specificity of the interactions. Immunoprecipitated proteins were resolved by SDS‒PAGE (8-12% gradient gels) and stained with Coomassie Brilliant Blue (1 h, RT). The bands were carefully excised from the gel and digested with sequencing-grade trypsin (37 °C, overnight). The peptide mixtures were separated on analytical C18 peptide trap columns (Zorbax 300SB; Agilent Technologies, Wilmington, DE). Mass spectrometry was conducted (Thermo Finnigan Q Exactive, San Jose, CA) in the 300.00-1800.00 m/z scan range. The raw MS data were transformed to mgf format and screened against the IPI Human v3.87 database (91,464 entries) with reversed decoys using SEQUEST HT software (Thermo Fisher Scientific, Waltham, MA) for identification with a 1% false discovery rate (FDR) threshold. Mass spectrometry services were provided by the Core Facility of Basic Medical Sciences of Shanghai Jiao Tong University School of Medicine.

The Gene Ontology (GO) enrichment analysis was conducted using Metascape 35 and KOBAS 36 to determine which particular pathways and biological processes were significantly associated with the list of identified proteins. The significance of the enrichment was evaluated through the hypergeometric test/Fisher's exact test, with multiple testing corrections applied utilizing the Benjamini and Hochberg method for FDR adjustment. Statistical computations were performed and plots were created using R software (v.4.2.2).

### Single-cell transcriptomic analysis of human testicular cells

Gene expression information from the Human Testis Development Atlas (GSE149512) 37 dataset was downloaded from the GSE database (NIH). Human testicular single-cell RNA sequencing datasets collected from five healthy adult donors were selected for analysis. Following quality control and normalization, the integrated datasets were processed using Seurat (v4.0), where gene expression matrices were scaled and subjected to principal component analysis (PCA). The top 20 principal components (PCs) were selected for downstream analytical steps, including the construction of a k-nearest neighbor (KNN) graph with refined edge weights based on shared nearest neighbor (SNN) modularity optimization. Unsupervised clustering was performed using the Leiden algorithm (resolution = 0.2), yielding 10 transcriptionally distinct cell populations. Cluster identities were annotated based on established marker genes (e.g., *DDX4* for germ cells and *SOX9* for Sertoli cells). Nonlinear dimensionality reduction was subsequently applied using t-distributed stochastic neighbor embedding (t-SNE) to visualize cellular distributions in two dimensions. Statistical computations were performed, and plots were created using R software (v.4.2.2). In addition, other single-cell expression data were obtained from the public databases, including the Human Protein Atlas (v25.0) 38 and the HumanTestisDB 39.

### Plasmid construction and transient transfection

Target genes were amplified from MDA-MB-453 or HEK293T cDNA using Phanta Flash Super-Fidelity DNA Polymerase (P510, Vazyme Biotech Co., Ltd., Nanjing, China) with designed primers based on the ELAPOR1 sequence (NM_020775.5) and the VPS54 sequence (NM_016516.3) obtained from the NCBI database. For proximity labeling, the BioID sequence was inserted in frame with ELAPOR1 using homologous recombination PCR. The PCR products were subsequently cloned and inserted into the p-Flag-CMV or pXJ40-HA vectors via restriction enzyme digestion and Gibson assembly (C116, Vazyme Biotech Co., Ltd., Nanjing, China). Corresponding full-length, truncated, or mutant constructs were generated and verified by Sanger sequencing.

HEK293T cells were cultured with Dulbecco's modified Eagle's medium (L110KJ, BasalMedia, Shanghai, China) supplemented with 1% penicillin‒streptomycin (C100C5, NCM Biotech, Suzhou, China) and 10% fetal bovine serum (Sigma, MO, USA) at 37 °C in a 5% CO_2_ incubator (Thermo Fisher Scientific).

Transient transfection was performed using polyethyleneimine (PEI) reagent (Sigma, MO, USA) according to the manufacturer's instructions. Briefly, DNA‒PEI complexes were formed in serum-free medium for 15 min before being applied to 70‒80% confluent cultures. Cells transfected with empty vectors served as negative controls. After transfection, the cells were incubated for 48‒72 h under standard culture conditions to maximize the recombinant protein yield.

### Generation of a stable ELAPOR1-overexpressing cell line

The ELAPOR1-Flag sequence was cloned and inserted into the lentiviral vector plasmid pCDH-CMV-GFP to generate a cell line stably expressing the ELAPOR1 protein. The ELAPOR1-Flag-GFP vector and the control empty GFP vector were transfected into HEK293T cells to obtain lentiviruses in the supernatants at 48-72 h through lentivirus packaging. The harvested supernatants were filtered and then concentrated using PEG8000 to isolate the viral particles. Viral particles and polybrene (10 μg/mL) were used to transduce target HEK293T cells. The infected cells were then cultured in complete DMEM supplemented with puromycin (1 μg/mL) for 7 days to screen for GFP-positive cells. The stable ELAPOR1-overexpressing cell line (ELAPOR1-Flag-GFP*) was validated by immunoblotting and compared to the empty vector-GFP*.

### Coimmunoprecipitation (co-IP)

Proteins were extracted as previously described. For the endogenous co-IP assay, 1-1.5 mg of testicular lysates and 10 µg of antibody were incubated overnight at 4 °C with rocking. An isotype-matched control IgG antibody was used as the negative control. Then, the protein complexes were captured by incubating the lysates with 50 µL of Protein A/G plus agarose (sc-2003, Santa Cruz Biotechnology, Texas, USA) with gentle inversion (3 h, 4 °C). The bead-antibody-antigen complexes were subsequently subjected to three rounds of washing with ice-cold lysis buffer containing protease inhibitors, followed by elution in 2 × loading buffer. For the exogenous co-IP assay, 1-1.5 mg of whole-cell lysates were incubated with anti-Flag affinity gel (A2220, Sigma, MO, USA; 20 μL bead volume), anti-HA agarose conjugate (26182, Pierce, IL, USA; 30 μL bead volume), or isotype IgG-Protein A/G plus agarose controls at 4 °C overnight. After the incubation, the immunocomplexes bound to the beads were purified by four washes with ice-cold lysis buffer before elution in 2 × loading buffer.

### Proximity labeling and capture of biotinylated proteins

For proximity labeling, cells were transfected with ELAPOR1-BioID plasmids or empty vectors. Afterward, the cells were incubated for 48 h with or without 50 µM biotin. Protein was extracted as previously described. The supernatants were collected and incubated with 30 µL of magnetic streptavidin beads (47503ES03, Yeasen, Shanghai, China) with gentle inversion (4 °C, overnight). After the incubation, the beads were collected on a magnetic stand and washed with the following series of buffers, as previously described 40: (1) twice with wash buffer 1 (2% SDS); (2) wash buffer 2 (500 mM NaCl, 1 mM EDTA, 0.1% deoxycholate, 1% Triton X-100, and 50 mM HEPES, pH 7.5); (3) wash buffer 3 (250 mM LiCl, 1 mM EDTA, 0.5% deoxycholate, 0.5% NP-40, and 10 mM Tris, pH 8.1); and (4) wash buffer 4 (50 mM NaCl, 50 mM Tris, pH 7.4). The proteins bound to the magnetic beads were eluted with 2 × loading buffer.

### Statistical analysis

Statistical graphs were generated using GraphPad Prism 9. All the statistical tests employed α = 0.05 as the significance threshold. The data are presented as the means ± SEMs, and p values are reported as follows: ns p ≥ 0.05, *p < 0.05, **p < 0.01, ***p < 0.001, and ****p < 0.0001. Statistical analyses were conducted using Student's t-test (unpaired, two-tailed) for two-group comparisons and one-way ANOVA for three-group comparisons. Specifics regarding sample sizes are provided in the figure legends.

## Results

### ELAPOR1 is highly expressed and colocalized with the acrosome during the early stage of acrosome biogenesis

**Figure 1:**
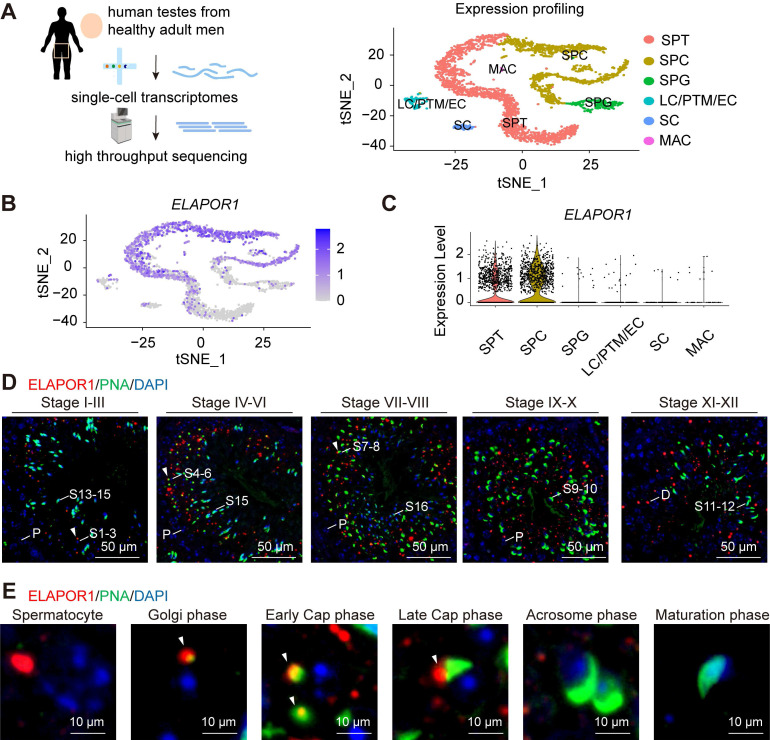
** ELAPOR1 is highly expressed in spermatocytes and spermatids. (A)** Single-cell analysis of human testes from healthy adult men. A t-distributed stochastic neighbor embedding (t-SNE) plot was constructed to illustrate the expression profile of the transcriptomes. SPG: spermatogonia; SPC: spermatocyte; SPT: spermatids/sperm; SC: Sertoli cell; LC: Leydig cell; PTM: peritubular myoid cell; EC: endothelial cells; MAC: macrophage. **(B)** t-SNE plot of the *ELAPOR1* expression pattern in specific cell types in the human testis. **(C)** Violin plot of *ELAPOR1* expression patterns in specific cell types in the human testis. **(D)** Immunofluorescence staining for ELAPOR1, peanut agglutinin (PNA) as a marker of acrosomes, and DAPI as a marker of nuclei in the mouse testis. White arrows indicate the colocalization of ELAPOR1 and PNA signals. P, pachytene spermatocytes; D, diplotene spermatocytes; S1-16, step 1-16 spermatids. Bar = 50 μm. **(E)** Representative magnified images of the staining of different stages of spermatids in **(D)** show the colocalization of ELAPOR1 with the early-stage acrosome (white arrows). Bar = 5 μm.

ELAPOR1 is highly expressed in the testes of adult humans according to several public databases, such as the Male Health Atlas, the Human Protein Atlas (Figure S1A), and the Human Testis DB (Figure S1B) 41. Interestingly, the single-cell sequencing analysis (Figure 1A) revealed that *ELAPOR1* is specifically enriched in spermatocytes and spermatids at the transcriptome level (Figure 1B-C), suggesting that it may play a role in spermiogenesis, especially during the early stage. The ELAPOR1 signal was not detected in mature spermatozoa or in spermatogonia (Figure S1C). Testicular sections from adult mice were analyzed by immunofluorescence staining with concurrent FITC-conjugated peanut agglutinin (PNA) to visualize the developing acrosomes and explore the spatial pattern of ELAPOR1 protein expression during spermatogenesis (Figure 1D). The images showed that ELAPOR1 is localized mainly in late-stage spermatocytes and the early-stage spermatids in mouse testes, as identified by the expression of PNA and the acrosomal vesicle protein ACRV1, which is consistent with the single-cell RNA-seq results (Figures 1D-E and S1D-E). Moreover, we found that ELAPOR1 specifically colocalized with proacrosomal vesicles in the Golgi phase and early cap phase (Figures 1D-E and S1D-E). Afterward, with the progression of acrosome maturation, the abundance of the ELAPOR1 protein decreased, and its colocalization gradually diminished. The developmental stage-restricted localization of ELAPOR1 suggests its transient yet critical function in establishing the acrosomal architecture. This spatiotemporally restricted expression pattern aligns with the well-accepted biological principle that early acrosome biogenesis necessitates precise coordination between gene expression windows and the spatial organization of organelle positioning 42, which ensures the proper sequence of molecular events required for acrosome formation.

### Germ cell-specific knockout of *Elapor1* leads to male infertility in mice

**Figure 2:**
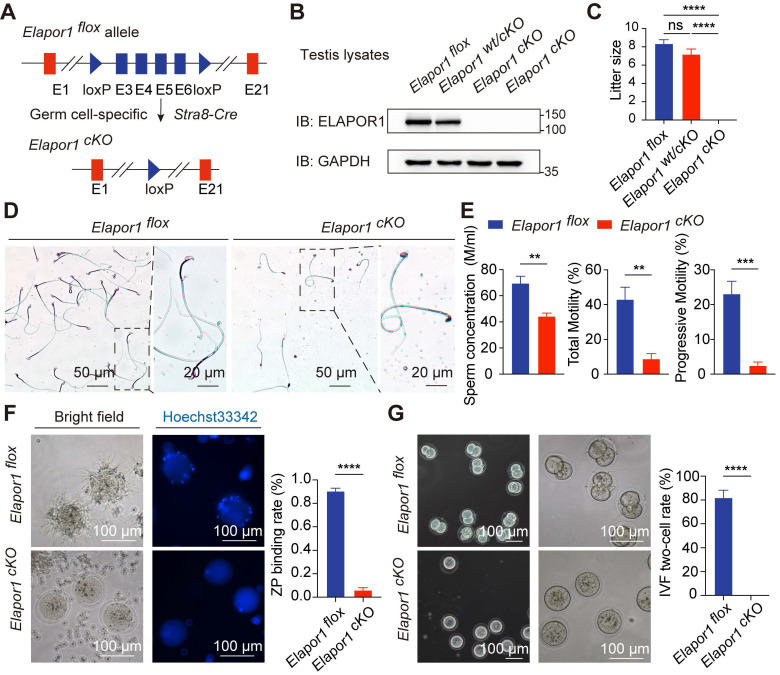
** Severe reproductive defects are observed in *Elapor1^cKO^* male mice. (A)** Graphical summary of the genetic engineering strategy for generating *Elapor1^flox^* and germ cell-specific *Elapor1^cKO^* mice. **(B)** Validation of the conditional knockout efficiency through an immunoblot analysis of ELAPOR1 protein levels in* Elapor1^flox^*, *Elapor1^wt/cKO^*, and *Elapor1^cKO^* testes. **(C)** Litter sizes obtained from *Elapor1^flox^*, *Elapor1^wt/cKO^*, and *Elapor1^cKO^* male mice mated with *Elapor1^flox^* female mice (n = 6). **(D)** Giemsa staining of sperm from *Elapor1^flox^* and *Elapor1^cKO^* mice. Bar = 50 μm in the main panels (left panels) and bar = 20 μm in the magnified panels (right panels). **(E)** Concentrations of total sperm, percentages of motile sperm, and percentages of progressive motile sperm in* Elapor1^flox^* and *Elapor1^cKO^* mice, as assessed via a computer-assisted semen analysis system (n = 6). **(F)** *In vitro* analysis of sperm-egg binding using sperm from adult *Elapor1^flox^* and *Elapor1^cKO^* mice. Nuclei were stained with Hoechst 33342. Bar = 100 μm. Zona pellucida (ZP) binding rates of sperm from *Elapor1^flox^* and *Elapor1^cKO^* mice were evaluated (n = 6). **(G)** Wild-type oocytes after 24 h of *in vitro* fertilization (IVF) with sperm from adult *Elapor1^flox^* and *Elapor1^cKO^* mice. Bar = 100 μm. Two-cell rates following the IVF of *Elapor1^flox^* and *Elapor1^cKO^* mice were evaluated (n = 6). The data are presented as the means ± SEMs. Statistical analyses were conducted using Student's t-test (unpaired, two-tailed) for comparisons between two groups or one-way ANOVA for comparisons among three groups. ns = no significant difference. ** P < 0.01; *** P < 0.001; and **** P < 0.0001.

With widespread cellular expression, ELAPOR1 mediates multiple cellular processes, as evidenced by diverse pathological manifestations upon its deletion, such as paligenosis, Baastrup's syndrome and estrogen excess 29, 43-45. Whole-body knockout of *Elapor1* has been previously reported to cause male infertility 29, 32, 33. We generated germ cell-specific *Elapor1* knockout mice (hereafter referred to as* Elapor1^cKO^*) using *Stra8-Cre* transgenic mice to verify whether the observed spermatogenic abnormalities originate from the testes autonomously rather than from secondary systemic effects following global *Elapor1* deficiency (Figure 2A). Consistent with our expectation, the germ cell-specific knockout effectively depleted the ELAPOR1 protein in *Elapor1^cKO^* mouse testis lysates compared with *Elapor1^wt/cKO^* and *Elapor1^flox^* mouse testis lysates, as shown by immunoblotting (Figures 2B and S2A).

*Elapor1^cKO^* mice, which were born at the expected Mendelian frequencies, presented no apparent developmental or physiological defects. We systematically compared the reproductive capacity of males of each genotype with that of *Elapor1^flox^* females through an 8-week breeding scheme. Homozygous* Elapor1^cKO^* mice exhibited complete infertility, whereas heterozygous *Elapor1^wt/cKO^* males maintained normal fertility comparable to that of *Elapor1^flox^* mice (Figure 2C). A morphological analysis of testes revealed comparable organ dimensions, weights and weight-to-body ratios between *Elapor1^cKO^* and *Elapor1^flox^* mice (Figure S2B).

Sperm were isolated from the cauda epididymis to assess the cause of the infertility in *Elapor1^cKO^* mice. Severe sperm malformations, such as an abnormal head shape, faulty neck structure and smaller head size, were observed (Figures 2D and S2C). The integrity of the *Elapor1^cKO^* sperm acrosome was impaired, as assessed by PNA staining to visualize the acrosomal membrane on sperm smears (Figure S2D). Taken together, the results revealed that *Elapor1^cKO^* sperm failed to undergo normal acrosomal remodeling and shaping and exhibited morphological defects similar to the hallmark traits of globozoospermia.

In addition to the abnormal morphology, the motility pattern of *Elapor1^cKO^* sperm was also defective under a microscope (Movies S1 and S2). According to CASA, *Elapor1^cKO^* mice exhibited a notable decrease in the sperm concentration, accompanied by significant impairments in motility and progressive movement capabilities (Figure 2E). We further evaluated the fertilization ability of *Elapor1^cKO^* sperm by assessing their functionality at different time points during the fertilization process, including the acrosome reaction (AR), sperm-egg binding, and early embryonic development. Following induction with the Ca²⁺ ionophore A23187, a large fraction of *Elapor1^cKO^* sperm retained PNA staining, indicating defective AR occurrence, which was significantly lower than that observed in control sperm (Figure S2E). Moreover, subsequent *in vitro* fertilization (IVF) assays demonstrated the impaired fertilization capacity of *Elapor1^cKO^* sperm, including a failure to bind to and penetrate the zona pellucida (Figures 2F and S2F). Consequently, *Elapor1^cKO^* sperm were unable to produce two-cell embryos (Figure 2G). These findings indicated that *Elapor1* deficiency led to impaired sperm-oocyte fusion and defects in fertilization, which may be the underlying causes of male infertility.

In conclusion, the results indicate that ELAPOR1 is critical for the proper morphology and fertilization capacity of mouse sperm, with its deficiency inducing male infertility and morphological defects similar to globozoospermia in humans.

### *Elapor1* germline knockout has no effect on Leydig or Sertoli cells

The expression of marker genes involved in spermatogenesis was quantified by real-time PCR (qRT‒PCR) and immunoblotting analysis in the testes of* Elapor1^flox^* and *Elapor1^cKO^* mice to further evaluate the effect of *Elapor1* germline knockout. The results revealed no significant alterations in either the transcript or protein levels of the representative functional markers in *Elapor1^cKO^* mouse testes (Figure S3A-D). However, our results revealed modest changes in the expression of specific genes, including decreased expression levels of *Neurog3* and increased expression levels of *Rara, Meioc, Sycp3* and *Prm2* in *Elapor1^cKO^* testes, suggesting that the deletion of *Elapor1* selectively affects specific stages or the progression of the spermatogenesis, rather than causing a global disruption.

Additionally, the structure and distribution of Sertoli cells were assessed by performing immunofluorescence staining for vimentin (Figure S3E) and SOX9 (Figure S3F). BTB integrity in *Elapor1^cKO^* mouse testes was normal, as revealed by the results of biotin tracer permeability assays (Figure S3G). Moreover, the tight junctions of the BTB were examined through immunofluorescence staining for Claudin-11 (Figure S3H) and ZO-1 (Figure S3I) and immunoblot analysis (Figure S3J). In conclusion, these results showed a limited effect of *Elapor1* knockout on other spermatogenic processes or on Leydig and Sertoli cells.

### ELAPOR1 deficiency leads to failure of the fusion of proacrosomal vesicles in malformed acrosomes

H&E-stained sections of testicular and epididymal tissues revealed morphological malformations in late spermatids and in the epididymal sperm of *Elapor1^cKO^* mice, suggesting that the absence of ELAPOR1 impaired morphogenesis and potential acrosomal disruptions in spermatid differentiation (Figure S4A-B). Considering that globozoospermia typically arises from impaired acrosome development, we performed a comparative immunofluorescence analysis of testicular tissue from *Elapor1^flox^* and *Elapor1^cKO^* mice using both PNA and acrosomal vesicle protein 1 (ACRV1) to systematically clarify the progression of acrosomal biogenesis (Figure S4C-D). In *Elapor1^flox^* mice, spermiogenesis followed the typical progression, beginning with the formation of a singular acrosomal vesicle from Golgi-derived proacrosomal vesicles, followed by acrosome flattening, nuclear laminae extension, and ultimately deposition onto the nuclear surface. Golgi-phase spermatids in *Elapor1^cKO^* mice maintained the expected spatial distribution pattern of the acrosome. However, during the cap phase, *Elapor1^cKO^* spermatids exhibited significant acrosomal abnormalities with discontinuous, punctate acrosomal structures distributed irregularly along the nuclear surface, in stark contrast to *Elapor1^flox^* spermatids (Figure S4C). This phenomenon persisted throughout the later stages of spermiogenesis, including sperm head remodeling and elongation, as indicated by irregular PNA staining patterns that revealed disorganized accumulation of acrosomal contents. Similar results were also observed for the ACRV1-marked acrosomal vesicle protein (Figure S4D). These results indicate that *Elapor1* knockout leads to significant defects in acrosomal biogenesis characterized by the structural disorganization of acrosomal vesicles, which becomes detectable as early as the cap phase of spermiogenesis.

**Figure 3:**
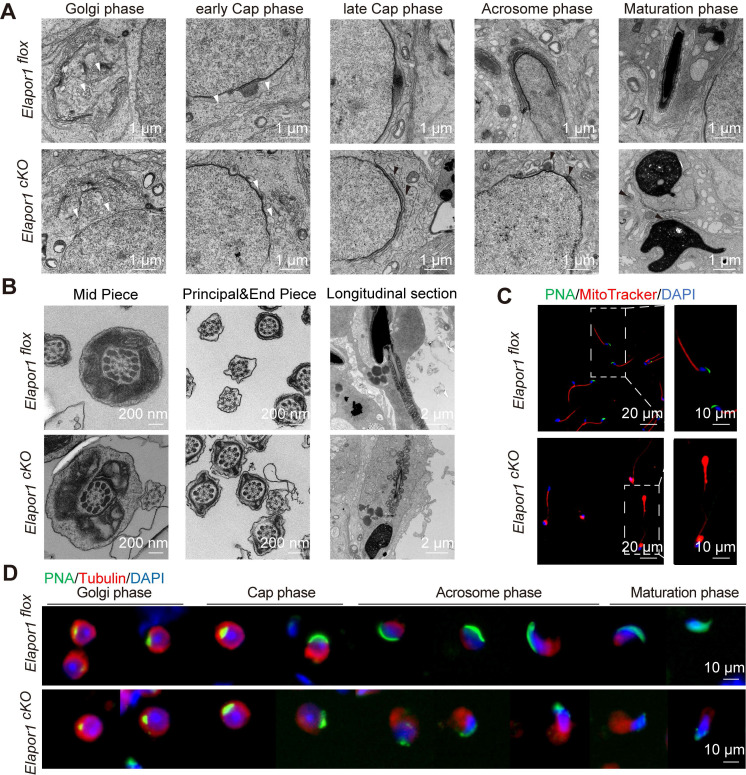
** ELAPOR1 is essential for proacrosomal vesicle fusion, mitochondrial organization, and manchette formation. (A)** Ultrastructural analysis of developing acrosomes in spermatids at different stages from *Elapor1^flox^* and *Elapor1^cKO^* mouse testes using transmission electron microscopy. Bar = 1 μm. White arrows, proacrosomal vesicles; black arrows, pseudoacrosome-like structures. **(B)** Ultrastructural analysis of *Elapor1^flox^* and *Elapor1^cKO^* spermatozoa in cross sections and longitudinal sections using transmission electron microscopy. Bar = 200 nm for cross sections, and bar = 2 μm for longitudinal sections. **(C)** Mitochondrial organization and acrosome structures of *Elapor1^flox^* and *Elapor1^cKO^* spermwere evaluated using MitoTracker and PNA staining. The nuclei were counterstained with DAPI. Bar = 20 μm in the main panels (left panels) and bar = 10 μm in the magnified panels (right panels). **(D)** Spermatids at different stages collected from *Elapor1^flox^* and *Elapor1^cKO^* mice were stained with PNA and α-tubulin to observe elongation of the manchette structures. The nuclei were counterstained with DAPI. Bar = 10 μm.

An ultrastructural analysis was performed using transmission electron microscopy (TEM) to elucidate the mechanism underlying the failure of acrosomal biogenesis. Consistent with the results of the immunofluorescence analysis, although *Elapor1^cKO^* spermatids maintained normal morphology of the Golgi apparatus and proacrosomal vesicle secretion was unaffected, they exhibited the abnormal accumulation of dispersed proacrosomal vesicles that failed to undergo the subsequent fusion required for proper acrosome formation during the cap phase (Figure 3A). In addition, a subset of *Elapor1^cKO^* spermatids exhibited abnormal acroplaxome curvature, indicating incomplete but detectable initiation of the assembly of the manchette, the specialized microtubule system that is required for the sperm head shape and elongation. Remarkably, the ultrastructural examination revealed the presence of a pseudoacrosomal structure characterized by a stratified membrane architecture closely spatially associated with the nuclear envelope, which has been consistently observed in some other mouse genetic models that exhibit globozoospermia-like phenotypes 18, 46.

Given that *Elapor1^cKO^* sperm showed severe motility defects, the sperm tail was also assessed in cross and longitudinal sections to reveal the mitochondria, axoneme microtubules, outer dense fibers (ODFs), and fiber sheaths. The ultrastructural analysis revealed that the mitochondria in the *Elapor1^cKO^* sperm tail displayed deformed structures and disrupted distributions (Figure 3B). Malformed sperm heads and acrosomes could also be clearly observed in the longitudinal sections of sperm from *Elapor1^cKO^* mice. In contrast, the flagellar axonemes of *Elapor1^cKO^* sperm maintained a typical 9 + 2 microtubular configuration, with well-aligned ODFs and well-defined dynein arms regularly positioned (Figure 3B). Furthermore, through the labeling of mitochondria with MitoTracker, the sperm from the cauda epididymides of *Elapor1^cKO^* mice exhibited severe mitochondrial disorganization, which suggests that the motility impairment of *Elapor1^cKO^* sperm may have arisen from the disordered mitochondria (Figure 3C). Moreover, the structural integrity of the manchette during spermiogenesis was examined using periodic acid-Schiff (PAS) staining and immunofluorescence staining for α-tubulin (Figures 3D and S5A-B). *Elapor1^cKO^* spermatids exhibited a disorganized and irregular manchette structure compared with the tightly organized microtubular array observed in control spermatids.

Taken together, these observations demonstrate impaired acrosome formation in *Elapor1^cKO^* mice spermatids, which is potentially due to defective vesicle fusion events during proacrosomal granule assembly. Disorganized mitochondria and malformed manchette during spermatogenesis may be responsible for the defective motility and aberrant head shape of *Elapor1^cKO^* sperm.

### ELAPOR1 is critical for acrosomal protein transport during spermatogenesis

**Figure 4:**
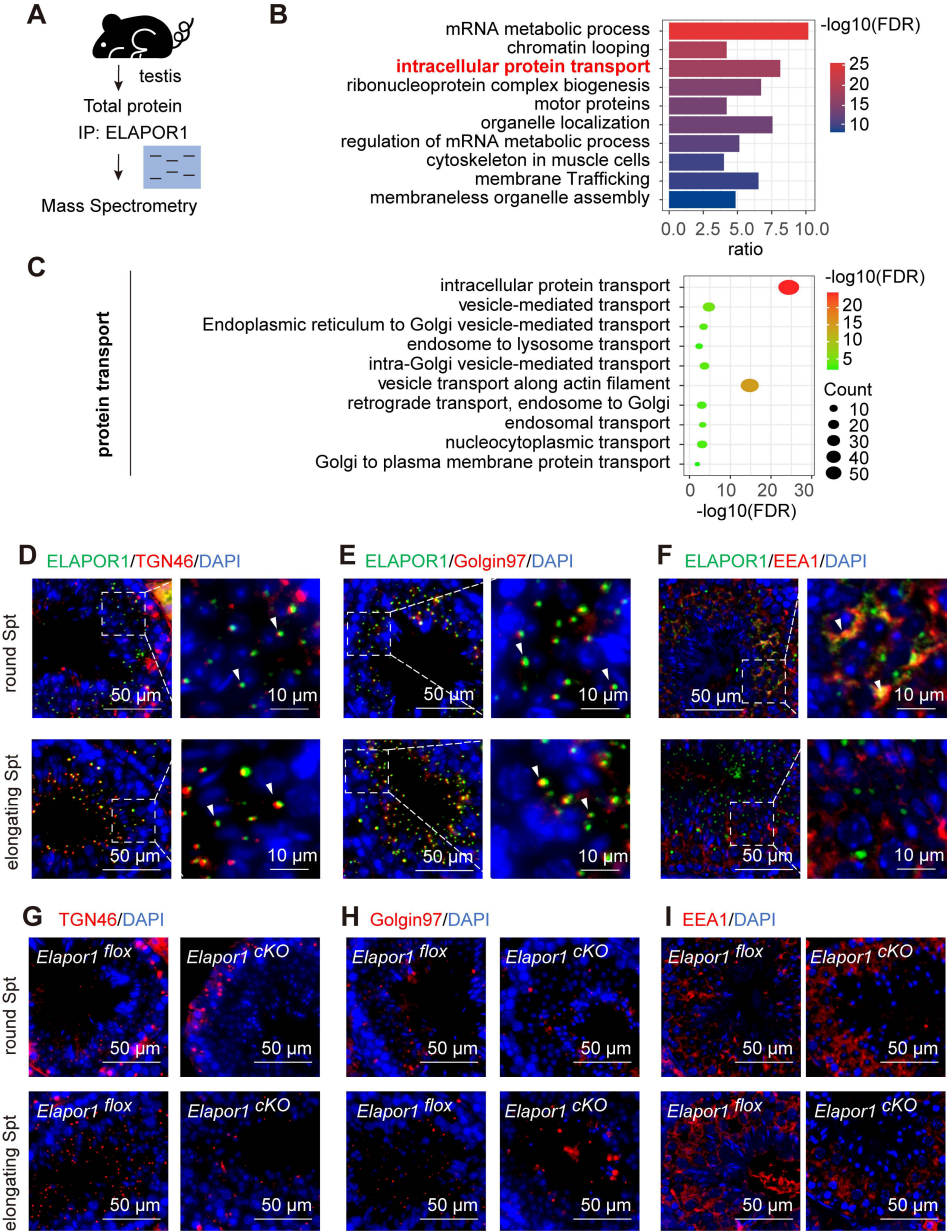
** ELAPOR1 regulates vesicular trafficking from the Golgi apparatus and the early endosomes in developing spermatids. (A)** Schematic diagram of mass spectrometry (MS) of *Elapor1^flox^* mouse testes. **(B)** Functional categorization of ELAPOR1-interacting proteins through a Gene Ontology (GO) enrichment analysis of MS data. The top 10 significantly enriched GO terms (Fisher's exact test, FDR-corrected) are displayed in a bar graph, with the color gradient representing the -log10(FDR) significance level. **(C)** Separate bubble plot of GO terms related to different types of protein transport categories. The top 10 significantly enriched genes are shown (Fisher's exact test, FDR-corrected), with the size of the bubble corresponding to the gene count and color gradient representing the -log10(FDR) significance level. **(D-F)** Immunofluorescence staining showing the colocalization (white arrows) of ELAPOR1 with trans-Golgi network integral membrane protein 2 **(TGN46, D)**, Golgin subfamily A member 1 **(Golgin97, E)**, and early endosome antigen **(EEA1, F)** in round and elongating spermatids (Spts). Bar = 50 μm in the main panels (left panels) and bar = 10 μm in the magnified panels (right panels). **(G-I)** Immunofluorescence staining for different organelle protein transport markers in round and elongating spermatids from *Elapor1^flox^* and* Elapor1^cKO^* mice. TGN46 is a marker for trans-Golgi network **(G)**, Golgin97 is a marker for endosome-Golgi vesicular transport **(H)**, and EEA1 is a marker for early endosomes **(I)**. Bar = 50 μm.

We investigated how ELAPOR1 regulates spermatogenesis at the molecular level by performing immunoprecipitation (IP) followed by mass spectrometry (MS) to identify and analyze the interactions in the testicular lysates of *Elapor1^flox^* mice (Figure 4A; Table S3). Functional annotation using Gene Ontology (GO) enrichment analysis revealed that ELAPOR1-interacting proteins were significantly involved in intracellular protein transport in the testis (Figure 4B). The enriched transport routes included Golgi and endosome-related protein transport, indicating that ELAPOR1 may play a vital role in these processes (Figure 4C). We investigated Golgi and endosome-related vesicle trafficking by performing immunofluorescence staining for distinct markers, including the trans-Golgi marker TGN46 (Figure 4D), the endosome-to-Golgi trafficking marker Golgin97 (Figure 4E), and the early endosome marker EEA1 (Figure 4F), in *Elapor1^flox^* mouse testes. Our results revealed the specific colocalization patterns of ELAPOR1 with these proteins, suggesting the potential involvement of ELAPOR1 in these vesicular transport processes during acrosome formation.

Furthermore, a comparative immunofluorescence analysis of *Elapor1^flox^* and *Elapor1^cKO^* mouse testicular sections revealed significant disruptions in the distribution patterns of the trans-Golgi network and early endosomes, which were scattered or missing in both round and elongating spermatids (Figure 4G-I). Additionally, a decreased TGN protein abundance in *Elapor1^cKO^* mouse testes was observed through immunofluorescence staining and immunoblotting (Figure S6A-B), indicating disruptions in the homeostasis of the trans-Golgi network. These results indicate that ELAPOR1 may be an essential factor in Golgi and endosome-related vesicle trafficking and appears to play a vital role in the proper transport of proacrosomal vesicles during acrosome biogenesis.

The intricate biogenesis of the acrosome involves proteins that contribute to four distinct developmental stages: the generation of proacrosomal vesicles, intracellular vesicle trafficking, vesicle coalescence, and acrosome nuclear anchoring 47. Although the acrosome primarily consists of Golgi-derived secretory vesicles, emerging evidence has demonstrated the involvement of components of the endocytic machinery in acrosome formation 48. Consequently, our findings lend substantial support to the hypothesis that both Golgi-derived vesicles and endosomal vesicles serve as critical sources of vesicular transport in acrosomal protein delivery.

### ELAPOR1 may interact with VPS54 and regulate its localization and assembly

**Figure 5:**
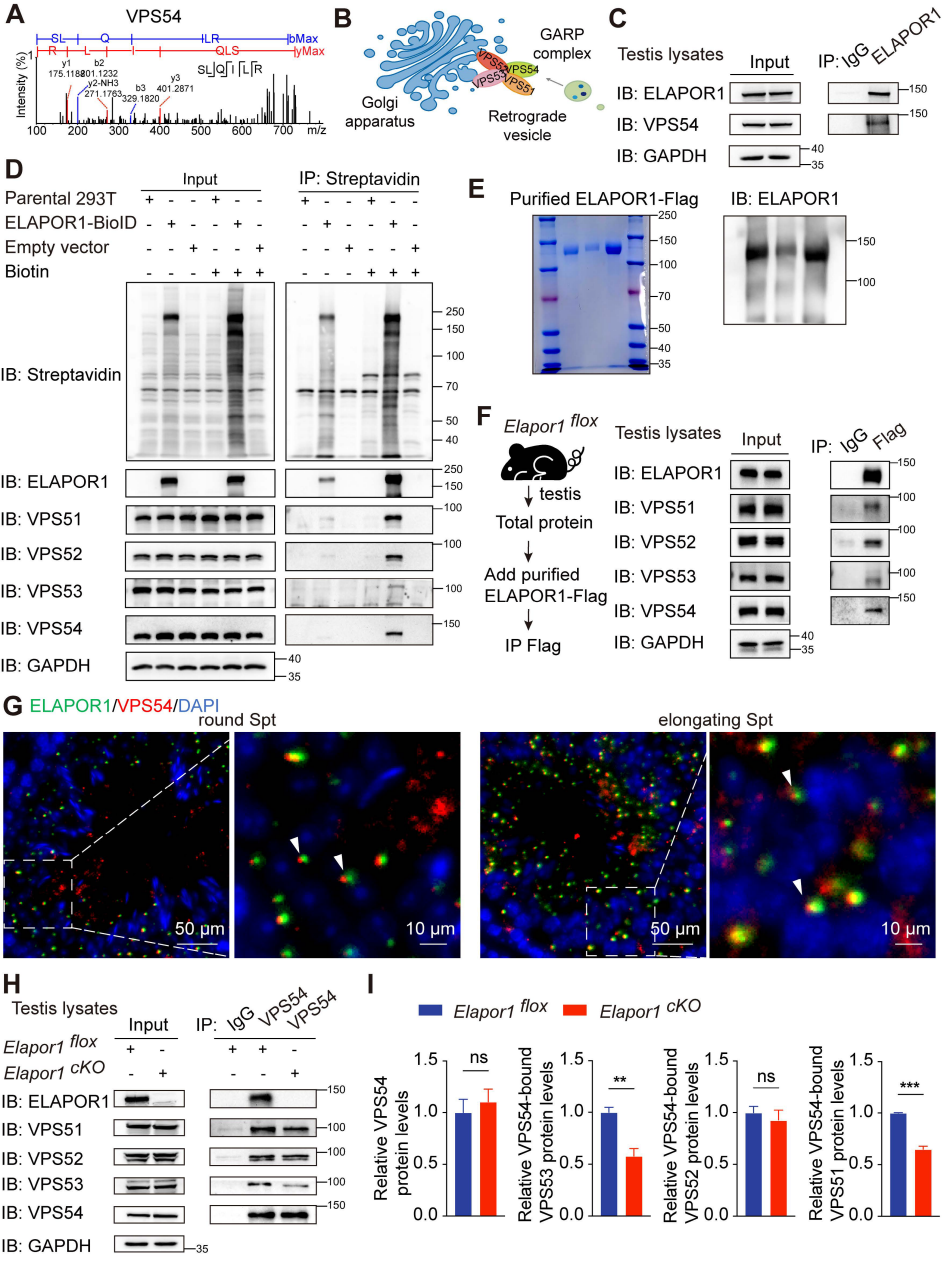
** ELAPOR1 interacts with VPS54 and the GARP complex. (A)** Fragment of the VPS54 peptide captured by MS. **(B)** Sketch of the GARP complex consisting of VPS54, VPS53, VPS52, and VPS51. **(C)** Co-IP of testicular lysates from *Elapor1^flox^* mice using an anti-ELAPOR1 antibody and an IgG control antibody. **(D)** Proximity labeling experiments using ELAPOR1-BioID in HEK293T cells incubated with 50 μM biotin. Untreated HEK293T cells (parental 293T cells), cells transfected with empty vectors, and cells incubated with PBS were used as control samples. Proteins biotinylated through BioID were subsequently immunoprecipitated using magnetic streptavidin beads and detected with HRP-streptavidin. **(E)** Purification of the ELAPOR1-Flag protein. The purity was assessed through Coomassie staining and immunoblot analysis. **(F)** Co-IP of purified ELAPOR1-Flag protein in testicular lysates of *Elapor1^flox^* mice using anti-Flag antibodies and IgG control antibodies. **(G)** Immunofluorescence staining showing the colocalization (white arrows) of ELAPOR1 with VPS54. Bar = 50 μm in the main panels (left panels) and bar = 10 μm in the magnified panels (right panels). **(H)** Co-IP of testicular lysates from *Elapor1^flox^* and *Elapor1^cKO^* mice using anti-VPS54 antibodies and IgG control antibodies. **(I)** Relative levels of the VPS53, VPS52, and VPS51 proteins bound to VPS54 in *Elapor1^flox^* and *Elapor1^cKO^* mouse testes (n = 3). The data are presented as the means ± SEMs. Statistical analyses were conducted using Student's t-test (unpaired, two-tailed) between the two groups. ns = no significant difference. ** P < 0.01; and *** P < 0.001.

Notably, our proteomic data revealed that ELAPOR1 is a novel interacting partner of VPS54 (Figure 5A). As a unique subunit of the Golgi-associated retrograde protein (GARP) complex, VPS54 mediates dynamic endosome-to-TGN vesicular trafficking (Figure 5B). An ELAPOR1-VPS54 interaction was observed in* Elapor1^flox^* mouse testicular lysates (Figure 5C). The protein‒protein interactions of ELAPOR1 with VPS54 and the GARP complex were validated by proximity labeling (Figure 5D) and coimmunoprecipitation (co-IP) assays (Figure S7A) in HEK293T cells. For further verification, the ELAPOR1-Flag protein was expressed and purified (Figure 5E) and subsequently added to *Elapor1^flox^* mouse testicular lysates for co-IP (Figure 5F). The results showed that the purified ELAPOR1-Flag protein could interact with components of the GARP complex, providing independent confirmation of their interactions. Immunofluorescence staining corroborated the colocalization of ELAPOR1 and VPS54 in spermatocytes and spermatids (Figure 5G), whereas the localization pattern of VPS54 in *Elapor1^cKO^* mice was disrupted (Figure S7B).

Furthermore, the interactions of VPS54 with the other subunits of the GARP complex, including VPS53 and VPS51, were significantly weakened in the testes of *Elapor1^cKO^* mice (Figure 5H-I). In contrast, the interaction of VPS54 with VPS53 was enhanced in stable ELAPOR1-overexpressing HEK293T cells (Figure S7C-D). These results suggest that ELAPOR1 may participate in the organization and assembly of the GARP complex. The VPS54-L967Q mutant maintains the ability to be incorporated into the GARP complex but is unstable and exhibits accelerated degradation, ultimately decreasing the protein levels of both the mutant VPS54 and the associated complex 49. Interestingly, the protein level of ELAPOR1 decreased, which was likely correlated with the reduced stability and abundance of the VPS54-L967Q mutant form (Figure S7E). Statistical analyses further revealed that the VPS54-L967Q mutation impaired the physical interaction between ELAPOR1 and VPS54 (Figure S7F). Furthermore, we investigated the expression patterns of the GARP subunits throughout the process of spermatogenesis by analyzing the single-cell RNA-seq database. The subunits of the GARP complex were specifically enriched in spermatocytes and spermatids, similar to ELAPOR1 (Figure S8A-B). In summary, these findings suggest that ELAPOR1 potentially interacts with VPS54 and the GARP complex, regulating its spatial organization and functional assembly during acrosome biogenesis. ELAPOR1 deficiency likely leads to disruptions in the sorting and recruitment of protein cargos essential for acrosome formation and vesicle fusion through its interaction with VPS54 and the GARP complex.

**Figure 6:**
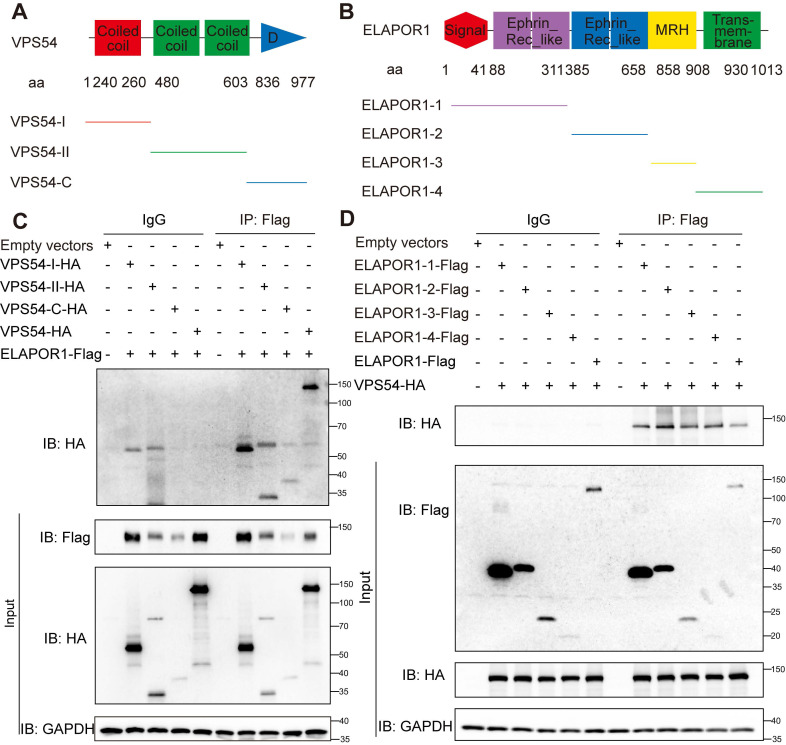
**ELAPOR1 and VPS54 have multiple binding sites that mediate their interaction. (A)** Functional domains of VPS54 and its truncation mutants. **(B)** Functional domains of ELAPOR1 and its truncation mutants. **(C)** Co-IP of lysates from HEK293T cells transfected with ELAPOR1-Flag and VPS54-truncated plasmids using anti-Flag antibodies and IgG control antibodies. Cells transfected with empty vectors were used as control samples. **(D)** Co-IP of lysates from HEK293T cells transfected with the VPS54-HA plasmid and ELAPOR1-truncated plasmids using anti-Flag antibodies and IgG control antibodies. Cells transfected with empty vectors were used as control samples.

Notably, VPS54 is structurally characterized by two functionally distinct domains: an N-terminal domain that facilitates its assembly into the GARP complex and a C-terminal domain that is crucial for mediating its specific localization to early endosomes 50. We constructed truncations of ELAPOR1 and VPS54 according to the functional domains to determine the exact regulatory function of the binding of ELAPOR1 and VPS54 (Figure 6A-B). Co-IP experiments revealed that ELAPOR1 and VPS54 likely have multiple binding sites that mediate their interaction (Figure 6C-D).

## Discussion

The acrosome, a specialized membrane-bound organelle unique to spermatozoa, plays an indispensable role in normal fertilization. Acrosome biogenesis begins with vesicle budding from the trans-Golgi network (TGN), followed by vesicle attachment to the acroplaxome, a cytoskeletal framework enveloping the sperm nucleus 42. These proacrosomal vesicles subsequently undergo fusion and eventually form the acrosome. Extensive research has demonstrated that abnormalities in critical stages of acrosome biogenesis, ranging from initial vesicle formation and intracellular trafficking to vesicular fusion events and culminating in the precise anchoring of the acrosome to nuclear structures, can potentially result in the development of globozoospermia. Considerable data have established that acrosome formation requires additional acrosome-specific components and factors derived from endocytic and exocytotic pathways. Notably, key components of the endocytic machinery, including EQTN 51, SPE39 52, UBP8 53, RGRF1 54 and VPS54 15, have been identified as critical proteins involved in acrosome biogenesis. In this study, we demonstrated that ELAPOR1 deficiency in murine germ cells caused severe male infertility, resulting in the production of spermatozoa with characteristic round heads and acrosome malformation, which recapitulated the hallmark features of human globozoospermia as previously described 7. Recent advances in genetic research have identified key mutations causing globozoospermia, providing potential pathways for improved diagnostics and targeted therapies. Our study suggests that ELAPOR1 could be regarded as a potential genetic risk factor for human infertility and may provide insights for future diagnoses or treatments of acrosome-associated diseases.

The defective acrosome formation in *Elapor1^cKO^* mice appears to primarily stem from the impaired fusion and coalescence of proacrosomal vesicles into a unified acrosomal structure. Molecular analyses revealed that these proteins are collectively involved in Golgi-associated processes and vesicular trafficking, suggesting that vesicle fusion failure in these mutants likely occurs at early stages of vesicle processing and formation, preceding the actual fusion event 18. Our immunofluorescence staining revealed that ELAPOR1 exhibits specific localization patterns within the Golgi/TGN, early endosomes, and proacrosomal structures during early acrosome development but is conspicuously absent from mature acrosomes, indicating its crucial role in vesicle formation and fusion processes during acrosome biogenesis. Moreover, *Elapor1^cKO^* sperm exhibited deformed mitochondria and an irregular manchette structure. Since mitochondria are essential for the ATP production required for flagellar motility, this abnormality likely underlies the observed impairment in *Elapor1^cKO^* sperm movement. In addition, given the established role of the manchette in shaping the nucleus and acrosome biogenesis, the disruption of this transient cytoskeletal structure may contribute to defective acrosome formation in *Elapor1^cKO^* mice. Additional molecular mechanisms may also be involved, which warrant further investigation.

Importantly, we elucidated a previously undescribed mechanism by which ELAPOR1 potentially facilitates acrosome formation through interactions with VPS54 and the GARP complex. Acting as a critical tethering factor, the GARP complex facilitates vesicle sorting and retrograde trafficking from endosomes to the TGN by stabilizing SNARE complex formation. This process is mediated by the N-terminal regions of VPS53 and VPS54 in conjunction with the conserved SNARE motifs 27. The binding of ELAPOR1 and VPS54 influences the assembly and stability of the GARP complex and further affects the tethering, trafficking, and fusion of proacrosomal vesicles during acrosome formation. These findings suggest that ELAPOR1 may play a critical role in the proper recruitment and fusion of vesicles through its interaction with the GARP complex. Notably, previous studies have shown that the depletion of GARP subunits impairs the retrograde transport of TGN46, resulting in markedly diminished TGN46 signaling at the TGN 26, 49. The distribution and abundance of TGN46 were also altered in this study, suggesting that ELAPOR1 may interact with the GARP complex to affect the maintenance of TGN homeostasis. Moreover, given the high expression levels of ELAPOR1 in various tissues, including the intestine, colon, and neurons, comprehensive investigations into the functions of ELAPOR1 in these systems would be worthwhile. In addition, the interaction between ELAPOR1 and VPS54 provides clues for further exploration of the physiological function of the GARP complex, especially in tissues with high ELAPOR1 expression. While we were submitting our study, a new article showed that ELAPOR1 could interact with STX12 and VAMP4 and thus regulate proacrosomal vesicle fusion 33, which confirms several findings in this study and further emphasizes the important role of ELAPOR1 in vesicle trafficking.

In summary, we found that germline-specific *Elapor1* is a critical adaptor protein that maintains the formation of the VPS54-mediated GARP complex during spermatogenesis. This regulatory function consequently facilitates the integration and precise fusion of proacrosomal vesicles, which is essential for proper acrosome formation. Our findings highlight the importance of the ELAPOR1-GARP complex in orchestrating the Golgi and endosome-related vesicle trafficking during spermatogenesis. These findings also advance the understanding of acrosome biogenesis and sperm function and could be beneficial for identifying potential diagnostic markers and therapeutic targets for future drug development.

## Supplementary Material

Supplementary figures, table and movie legends.

Supplementary table 1.

Supplementary table 2.

Supplementary table 3.

Supplementary movie 1.

Supplementary movie 2.

## Data Availability

The research data supporting the findings of this study are available from the corresponding author upon reasonable request.

## Abbreviations

ELAPOR1: endosome/lysosome-associated apoptosis and autophagy regulator 1
VPS54: vacuolar protein sorting-associated protein 54
TGN46: trans-Golgi network integral membrane protein 2
ACRV1: acrosomal vesicle protein1
EEA1: early endosome antigen 1
Golgin97: Golgin subfamily A member 1
GARP: Golgi-associated retrograde protein
PBS: phosphate buffer saline
CASA: computer-assisted semen analysis
TEM: transmission electron microscopy
RT‒qPCR: reverse transcription quantitative polymerase chain reaction
H&E: hematoxylin and eosin
IF: immunofluorescence
PNA: peanut agglutinin
DAPI: 4,6-diamidino-2-phenylindole
MS: mass spectrometry
GO: Gene Ontology
FDR: false discovery rate
scRNA-seq: single cell RNA sequencing
PCA: principal component analysis
tSNE: t-distributed stochastic neighbor embedding
SSC: spermatogonial stem cell
SPG: spermatogonia
SPC: spermatocyte
SPT: spermatids/sperm
SC: Sertoli cell
LC: Leydig cell
PTM: peritubular myoid cell
EC: endothelial cells
MAC: macrophage
IgG: immunoglobulin G
*Elapor1^cKO^*: *Elapor1^flox/-^;Stra8-Cre*
*Elapor1^wt/cKO^*: *Elapor1^wt/flox^*;*Stra8-Cre*
*Elapor1^flox^*: *Elapor1^flox/flox^*
