# Supplementary material for: ELAPOR1 regulates VPS54-mediated GARP complex formation and proacrosomal vesicle fusion during spermatogenesis
Source: Theranostics. 2026 Mar 25;16(10):5571–88. doi: 10.7150/thno.131535 (PMC13080796; doi:10.7150/thno.131535)
Supplement: Supplementary file 1 — Supplementary figures, table and movie legends. [file thnov16p5571s1.pdf]

Supplementary figures and materials legends

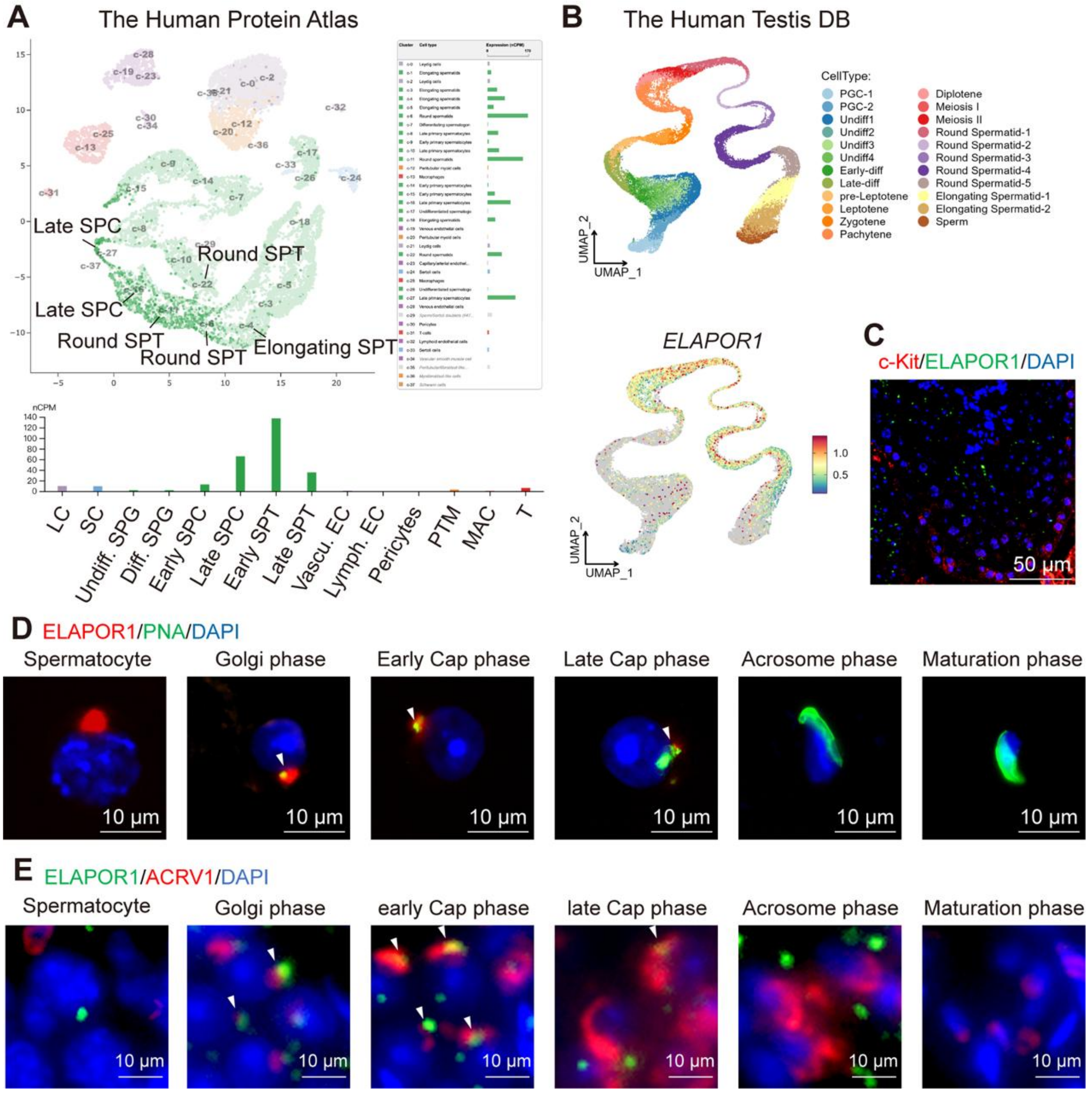

Figure S1. Additional evidence for ELAPOR1 expression in spermatocytes and spermatids. (A) Single-cell RNA expression data for *ELAPOR1* in the cell type clusters identified in the human testis from the Human Protein Atlas database, visualized by a UMAP plot and bar plots. The labels on the UMAP plot indicated cell-type clusters with RNA Expression (nCPM) > 50. Late SPC: Late primary spermatocyte; Round SPT: Round spermatids; Elongating SPT: Elongating spermatids; LC: Leydig cell; SC: Sertoli cell; Undiff. SPG: Undifferentiated spermatogonia; Diff. SPG: Differentiated spermatogonia; Early SPC: Early primary spermatocyte; Early SPT: Early spermatids; Late SPT: Late spermatids; Vascu. EC: Vascular endothelial cells; Lymph. EC: Lymphatic endothelial cells; PTM: peritubular myoid cells; MAC: macrophages; T: T-cells. (B) Single-cell RNA expression data for *ELAPOR1* in the cell type clusters identified in the human testis from the Human Testis DB, visualized by UMAP plots. (C) Immunofluorescence staining of ELAPOR1 and

c-Kit (Kit proto-oncogene receptor tyrosine kinase) in *Elapor1*^{flox} mouse testes. Bar = 50 μm. (D) Immunofluorescence staining for ELAPOR1, PNA as a marker of acrosomes, and DAPI as a marker of nuclei in isolated spermatocytes and spermatids from the mouse testes. Bar = 10 μm. White arrows indicate the colocalization of ELAPOR1 and PNA signals. Bar = 10 μm. (E) Immunofluorescence staining showing the colocalization (white arrows) of ELAPOR1 with acrosomal vesicle protein 1 (ACRV1). Bar = 10 μm.

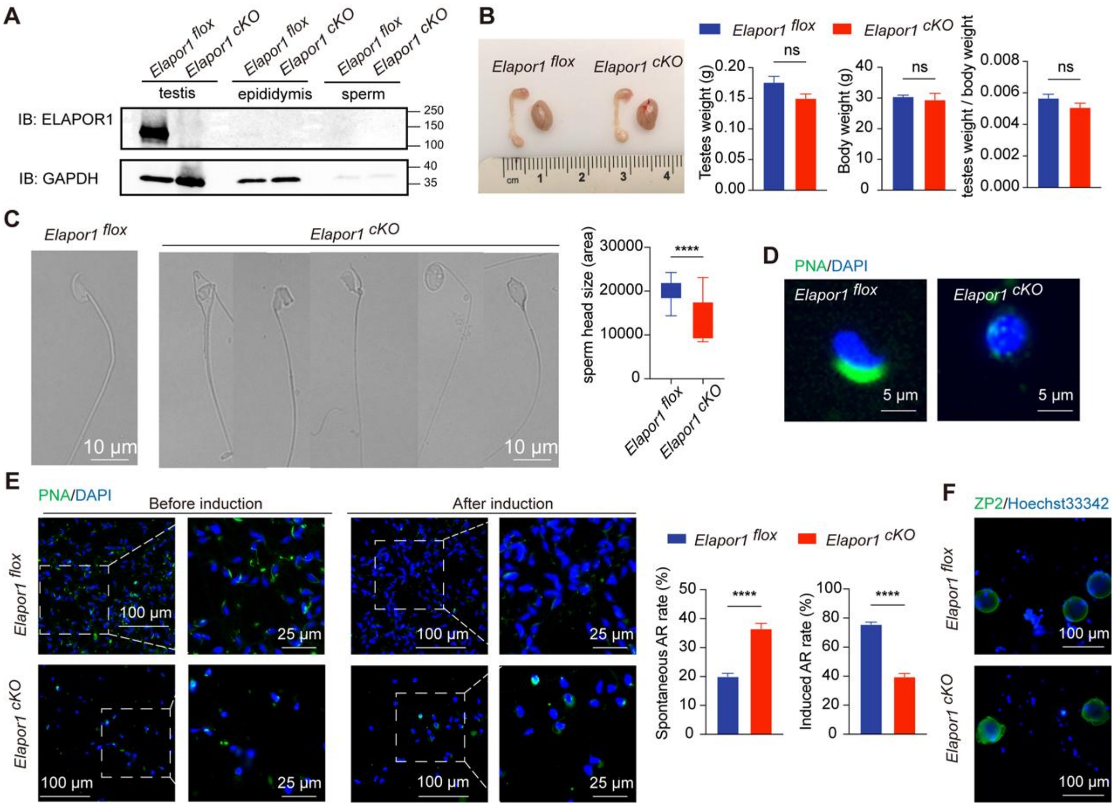

Figure S2. ELAPOR1 deficiency leads to nonfunctional acrosomes. (A) Immunoblot analysis of ELAPOR1 protein levels in testes, epididymis, and sperm from *Elapor1*^{flox} and *Elapor1*^{cKO} mice. (B) Morphological images of the testes from *Elapor1*^{flox} and *Elapor1*^{cKO} mice. Average individual testes weights, body weights, and testes/body weights of *Elapor1*^{flox} and *Elapor1*^{cKO} mice (n = 3). (C) Representative images of sperm from *Elapor1*^{flox} and *Elapor1*^{cKO} mice. Bar = 10 μm. The sperm head sizes from *Elapor1*^{flox} and *Elapor1*^{cKO} mice (n = 28). (D) Fluorescence staining of the acrosome with PNA of sperm from *Elapor1*^{flox} and *Elapor1*^{cKO} mice. Bar = 5 μm. (E) Acrosome reaction assessed by PNA staining of sperm from *Elapor1*^{flox} and *Elapor1*^{cKO} before and after A23187 induction. Nuclei were stained with DAPI. Bar = 100 μm in the main panels (left panels) and bar = 25 μm in the magnified panels (right panels). Spontaneous acrosome reaction rates and induced acrosome reaction rates of *Elapor1*^{flox} and *Elapor1*^{cKO} sperm (n = 6). (F) Fluorescence staining for ZP2 (pellucida sperm-binding protein 2) of eggs after binding with sperm from adult *Elapor1*^{flox} and *Elapor1*^{cKO} mice. Nuclei were stained with Hoechst 33342. Bar = 100 μm. The data are presented as the means ± SEMs. Statistical analyses were conducted using Student's t-test (unpaired, two-tailed) for comparisons between two groups. ns = no significant difference. **** $P < 0.0001$.

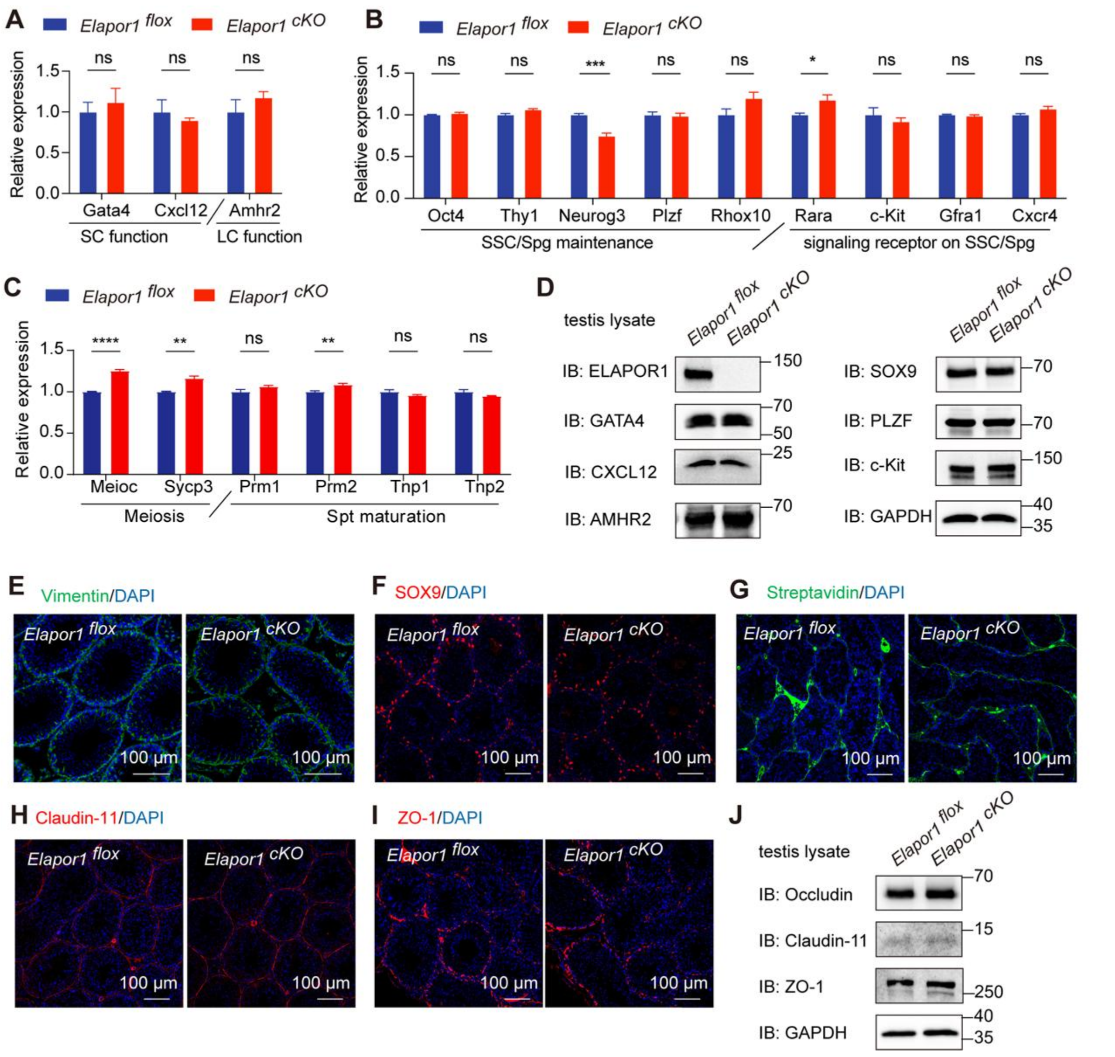

Figure S3. The impact of ELAPOR1 knockout on other spermatogenic processes and the integrity of the blood–testis barrier in the testes.

(A–C) RT–qPCR-based validation of the expression patterns of spermatogenic marker genes in *Elapor1^{flox}* and *Elapor1^{cKO}* mouse testes (n = 4). SC: Sertoli cell; LC: Leydig cell; SSC, spermatogonial stem cell; Spg, spermatogonia; Spt, spermatids/sperm. (D) Immunoblot analysis of protein levels of spermatogenic marker genes in testicular lysates from *Elapor1^{flox}* and *Elapor1^{cKO}* mice. (E–F) Immunofluorescence staining of Vimentin as a marker of blood–testis barrier (E) and SOX9 as a marker of Sertoli cells (F) in *Elapor1^{flox}* and *Elapor1^{cKO}* mouse testes. Bar = 100 µm. (G) Immunofluorescence staining of Streptavidin-488 showing the permeability through the blood–testis barrier in the seminiferous tubules from *Elapor1^{flox}* and *Elapor1^{cKO}* mice by a biotin tracer 30 min after injection. Bar = 100 µm. (H–I) Immunofluorescence staining for Claudin-11 (H) and ZO-1 (I) as markers of tight junctions in *Elapor1^{flox}* and *Elapor1^{cKO}* mouse testes. Bar = 100 µm. (J)

Immunoblot analysis of protein levels of marker genes of tight junctions in testicular lysates of *Elapor1*^{flox} and *Elapor1*^{cKO} mice. The data are presented as the means ± SEMs. Statistical analyses were conducted using Student's t-test (unpaired, two-tailed) for comparisons between two groups or one-way ANOVA for comparisons among three groups. ns = no significant difference. *$P < 0.05$; ** $P < 0.01$; *** $P < 0.001$; and **** $P < 0.0001$.

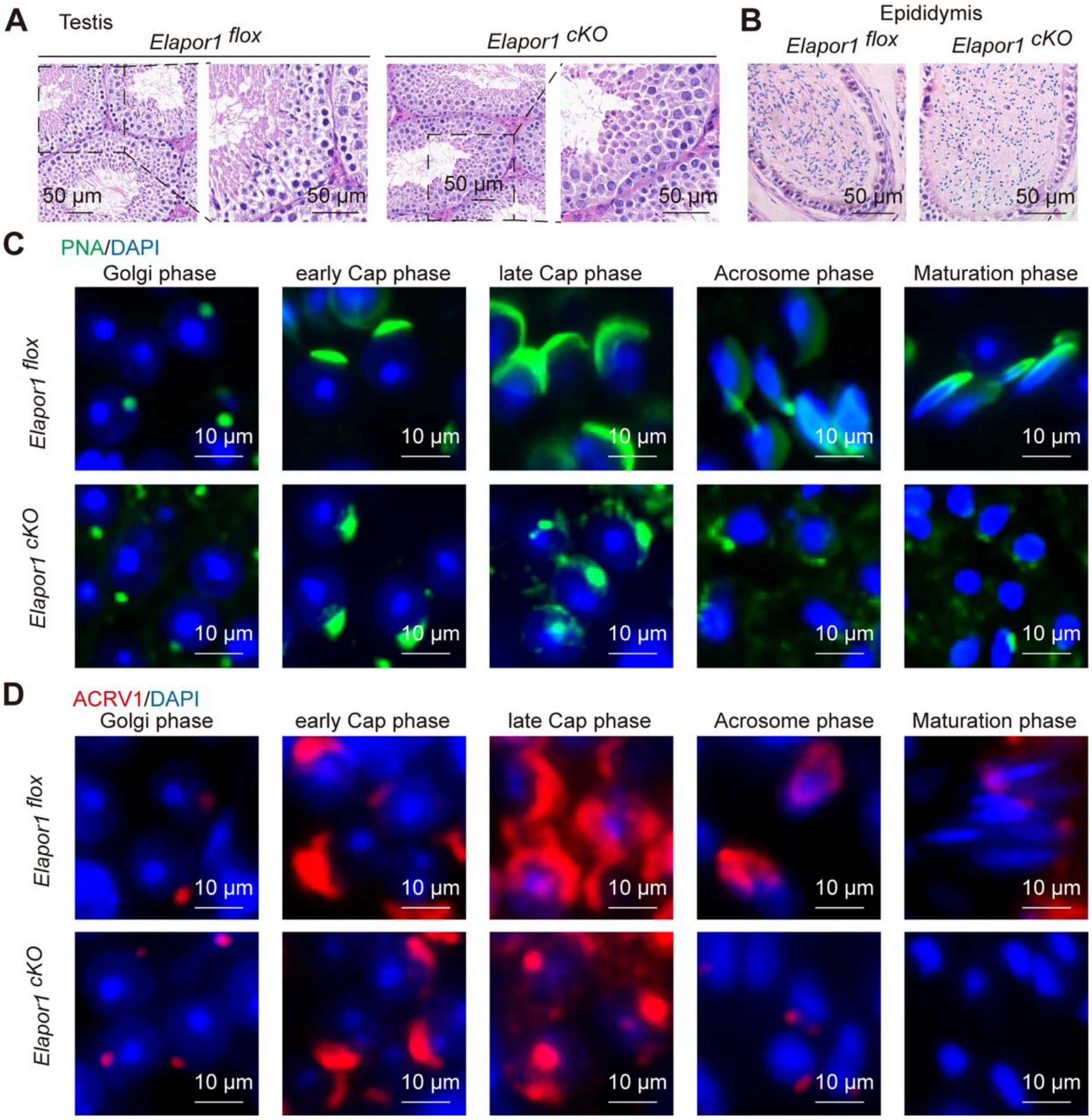

Figure S4. The abnormal morphology and defective acrosome formation of *Elapor1*^{cKO} sperm.

(A–B) H&E staining of the testicular (A) and epididymal (B) sections from *Elapor1*^{flox} and *Elapor1*^{cKO} mice. Bar = 50 μm. (C–D) Fluorescence staining of adult testis sections from *Elapor1*^{flox} and *Elapor1*^{cKO} mice with PNA (C), ACRV1 (D), and DAPI. Bar = 10 μm.

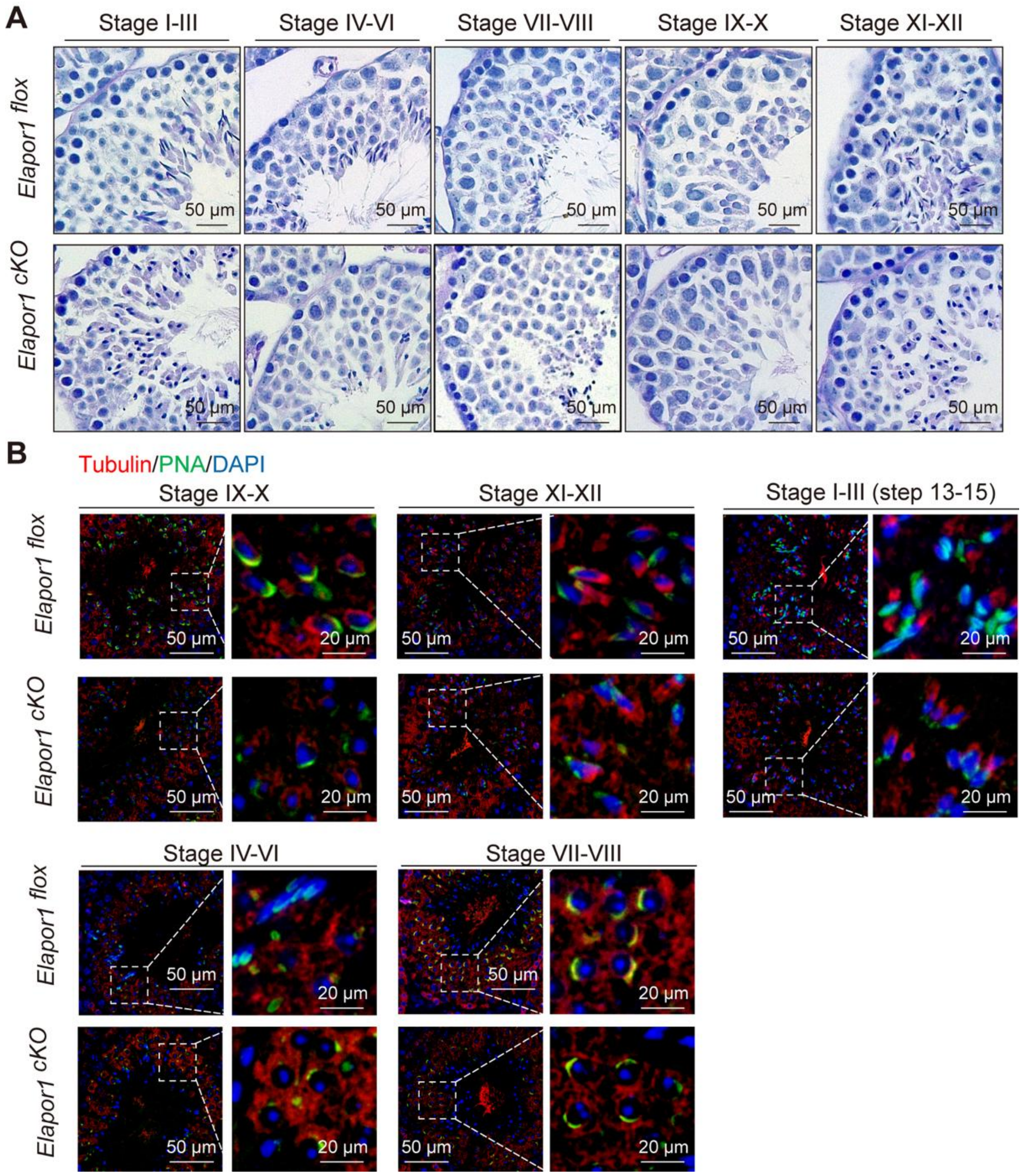

Figure S5. ELAPOR1 knockout disrupts the manchette during spermatogenesis. (A) The Periodic acid–Schiff (PAS) staining of seminiferous tubules of different stages from *Elapor1*^{flox} and *Elapor1*^{cKO} mice. Bar = 50 μm. (B) Immunofluorescence staining of tubulin as a marker of manchette formation, PNA as a marker of acrosome formation, and DAPI as a marker of nuclei in *Elapor1*^{flox} and *Elapor1*^{cKO} mouse testes. Bar = 50 μm in the main panels (left panels) and bar = 20 μm in the magnified panels (right panels).

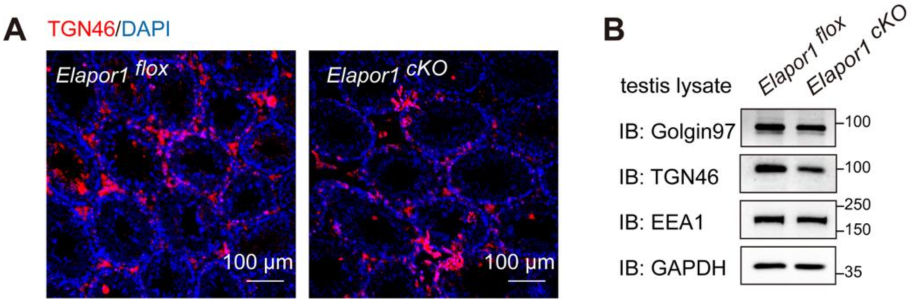

Figure S6. The disruptions of the TGN in *Elapor1*^{cKO} mouse testes. (A) A bigger panel of representative immunofluorescence staining images of TGN46 in the testes from *Elapor1*^{flox} and *Elapor1*^{cKO} mice. Bar = 100 μm. (B) Immunoblot analysis of protein levels of vesicle transport marker genes in testicular lysates from *Elapor1*^{flox} and *Elapor1*^{cKO} mice.

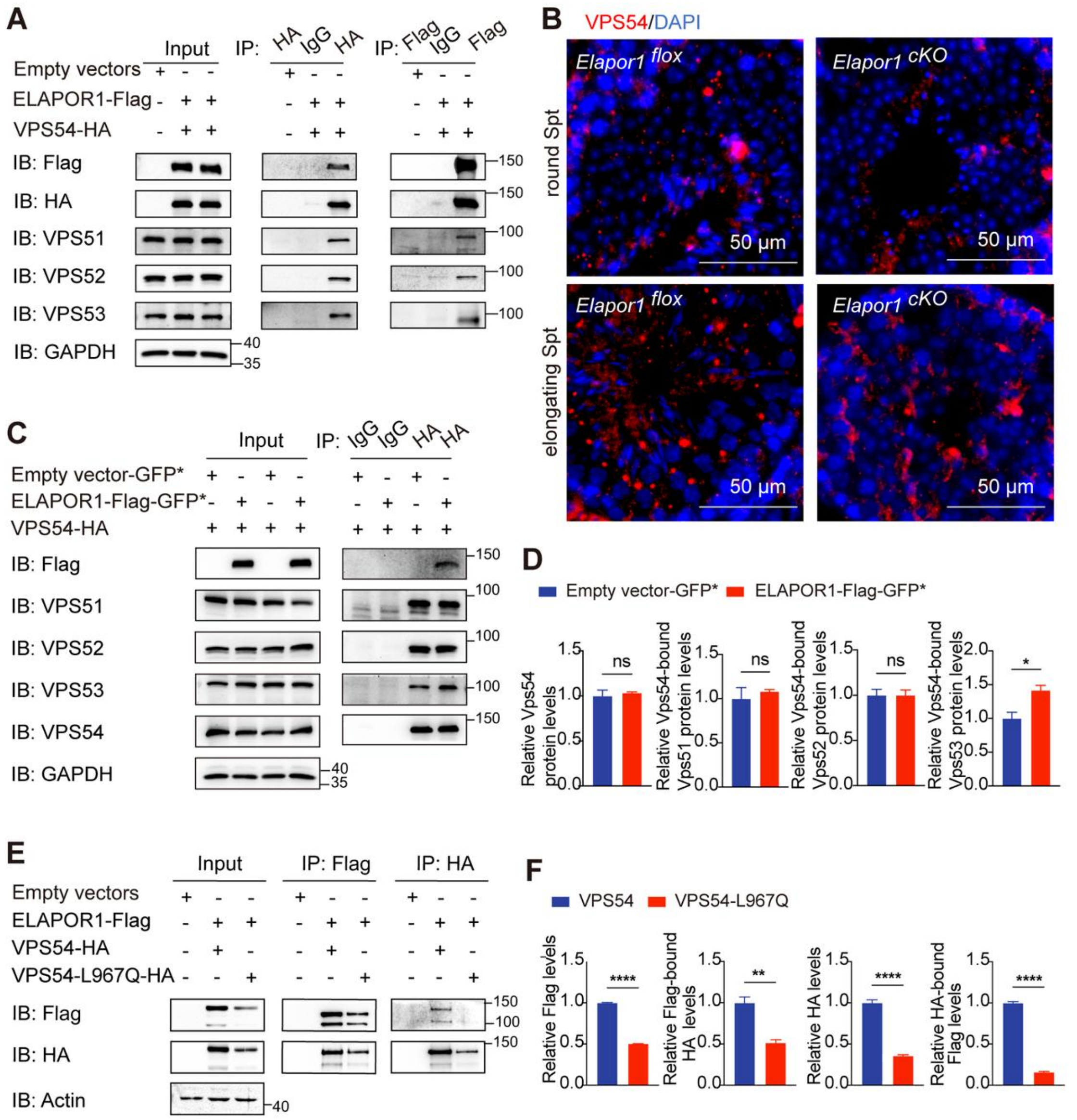

Figure S7. ELAPOR1 regulates the GARP complex assembly through interaction with VPS54. (A) Co-IP of lysates from HEK293T cells transiently transfected with ELAPOR1-Flag and VPS54-HA plasmids using anti-Flag antibodies, anti-HA antibodies, and IgG control antibodies. Cells transfected with empty vectors were used as control samples. (B) Immunofluorescence staining of ELAPOR1 and VPS54 in round and elongating spermatids in *Elapor1*^{flox} and *Elapor1*^{cKO} mouse testes. Bar = 50 μm. (C) Co-IP of lysates from HEK293T cell lines that constitutively expressed ELAPOR1-Flag-GFP* protein and were transfected with VPS54-HA plasmids using anti-HA antibodies and IgG control antibodies. Cell lines that constitutively expressed empty vectors-GFP* and were transfected with VPS54-HA plasmids were used as control samples. (D) Relative levels of VPS53, VPS52, and VPS51 proteins bound toVPS54 were evaluated (n = 3). (E) Co-IP of lysates

from HEK293T cells transfected with ELAPOR1-Flag and VPS54-HA or VPS54-L967Q-HA mutant plasmids. Cells transfected with empty vectors were used as control samples. (F) Relative levels of HA bound to Flag and Flag bound to HA ($n = 3$). The data are presented as the means ± SEMs. Statistical analyses were conducted using Student's t-test (unpaired, two-tailed) for comparisons between two groups or one-way ANOVA for comparisons among three groups. ns = no significant difference. *$P < 0.05$; ** $P < 0.01$; and **** $P < 0.0001$.

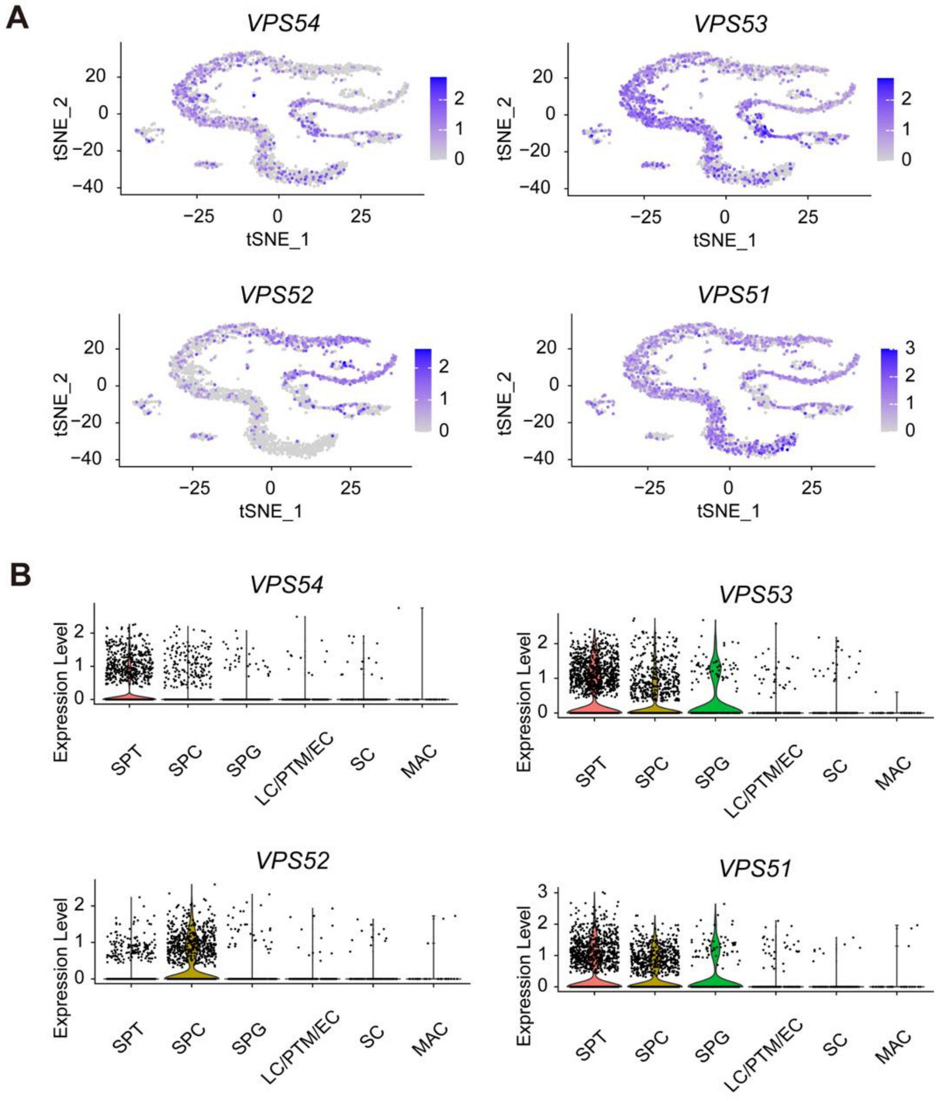

Figure S8. The expression patterns of the GARP complex subunits from human testes. (A) t-SNE plots of the expression patterns of *VPS54*, *VPS53*, *VPS52*, and *VPS51* in specific cell types from human testes. (B) Violin plots of the expression patterns of *VPS54*, *VPS53*, *VPS52*, and *VPS51* in specific cell types from human testes.

Movie S1. Video of sperm swimming capacity from *Elapor1^{flox}* mice. Representative movie showing motility patterns of *Elapor1^{flox}* mouse sperm.

Movie S2. Video of sperm swimming capacity from *Elapor1^{cKO}* mice. Representative movie showing motility patterns of *Elapor1^{cKO}* mouse sperm.

Table S1. Resources list.

Table S2. Primer list.

Table S3. Identified ELAPOR1 interacting protein list by MS.
